# miR-146b/Btg2 axis as a potential inducer of islet beta-cell decline during the progression of obesity to T2DM

**DOI:** 10.1016/j.gendis.2025.101621

**Published:** 2025-04-02

**Authors:** Weixuan Wang, Dan Ma, Yong Chen, Rui Cheng, Ting Zhang, Qian Ge, Xi Li

**Affiliations:** a The Institute of Life Sciences, School of Basic Medicine, Chongqing Medical University, Chongqing 400016, China; b Department of Endocrinology, First Affiliated Hospital of Chongqing Medical University, Chongqing 400016, China; c Department of Hepatobiliary Surgery, First Affiliated Hospital of Chongqing Medical University, Chongqing 400016, China; d Center of Obesity and Metabolic Diseases, Department of General Surgery, The Third People's Hospital of Chengdu, Affiliated Hospital of Southwest Jiaotong University & the Second Affiliated Hospital of Chengdu, Chongqing Medical University, Chengdu, Sichuan 610031, China

**Keywords:** Apoptosis, Beta-cell, Cell proliferation, Islet, microRNA, Obesity, Type 2 diabetes mellitus

## Abstract

We evaluated the potential mechanisms responsible for inducing beta-cell decline during the progression of obesity to type 2 diabetes mellitus (T2DM). Between February 2021 and February 2022, 25 subjects with non-diabetic obesity, 20 subjects with obesity and new-onset T2DM, and 25 healthy volunteers were recruited. Circulating exosome-contained miRNA expression profiling was performed by miRNA sequencing. The role of specific miRNA was analyzed by a gain-of-function approach in Min6 beta-cells, mouse islets, and human islets. Expression of 83 exosomal miRNAs was differently regulated in the circulation of subjects with non-diabetic obesity. We focused on miR-146b, which was mildly up-regulated in non-diabetic obesity and dramatically up-regulated in obese new-onset T2DM. Using an obese diabetic *db/db* mouse model, we found the expression of miR-146b to be mainly increased in islets. Overexpression of miR-146b in mouse beta-cells, mouse islets, and human islets *in vitro* facilitated beta-cell apoptosis yet inhibited its proliferation and insulin synthesis, leading to impaired insulin secretion. Eventually, miR-146b directly targeted the B cell translocation gene 2 (*Btg2*), an antiapoptotic transcriptional factor. Overexpression of *Btg2* reversed miR-146b-induced apoptosis and -suppressed proliferation in beta-cells. miR-146b that targets *Btg2* might be a predictive biomarker and an inducer of beta-cell decline.

## Introduction

Obesity is a substantial risk factor for developing type 2 diabetes mellitus (T2DM). About one-third of obese individuals develop T2DM.^1^^,^^2^ The progressive decline in islet beta-cell mass and function plays a crucial role in the natural history of obesity to T2DM. In adults, beta-cell mass is controlled by number (proliferation, neogenesis, and apoptosis) and size (hypertrophy). Reduced beta-cell number (possibly induced by increased apoptosis) rather than size is a major determinant of beta-cell loss and the onset of T2DM in humans.^3^, ^4^, ^5^ Yet, the signals controlling beta-cell survival are not fully understood in the context of disease progression. It has been proposed that mitochondrial dysfunction, oxidative and endoplasmic reticulum stress, dysfunctional triglyceride/free fatty acid cycling, and lipotoxicity in obesity could trigger alterations in the level of key genes as well as microRNAs (miRNAs).^6^ miRNAs are small non-coding RNAs that regulate gene expression by inducing target mRNA degradation. Each miRNA can have hundreds of targets, thereby triggering pleiotropic effects in cells.^7^ Moreover, miRNAs are encapsulated in exosomes, a type of extracellular vesicle, and hence protected from degradation in blood. Compared with free miRNAs, circulating exosomal miRNAs are more stable and can potentially be used as diagnostic biomarkers. They can also be taken up by neighboring or distant cells, and subsequently modulate the recipient cells.^8^ Since their discovery, miRNAs have been revealed to play significant roles in a wide spectrum of diseases, including T2DM.^9^ Emerging evidence has identified miRNAs as essential regulators of beta-cell physiology.^10^ The dysregulation of miRNAs through Dicer1 (an essential RNase for active miRNA biogenesis) ablation in early embryonic pancreatic progenitor cells in mice results in severe deficiencies in the formation of beta-cells and the establishment of diabetes within 2 weeks of birth.^11^^,^^12^ Likewise, deletion of Dicer1 in beta-cells leads to loss of insulin expression and the development of diabetes in adult mice.^13^

In this study, we aimed to identify the potential mechanisms responsible for inducing beta-cell decline during the progression of obesity to T2DM. We first evaluated the exosomal miRNA expression profile in the circulation of subjects with non-diabetic obesity as well as subjects with obesity and new-onset T2DM. Second, we characterized the function of miR-146b, which was mildly up-regulated in obesity and dramatically up-regulated in newly established T2DM.

## Materials and methods

### Subjects

Between February 2021 and February 2022, 25 subjects with non-diabetic obesity and 20 subjects with obesity and new-onset T2DM who visited the Obesity Clinic in our hospital were recruited. The subjects with new-onset T2DM were defined as they were newly diagnosed and not yet treated with diabetic drugs. Another 25 healthy volunteers (with healthy weight and matched for age and sex) were recruited as the control group. Body mass index ≥ 28 kg/m^2^ corresponds to obesity, and 18.5 kg/m^2^ ≤ body mass index < 24 kg/m^2^ corresponds to healthy weight. Diabetes was diagnosed based on an oral glucose tolerance test with fasting blood glucose level ≥ 7.0 mmol/L and 2-h glucose level ≥ 11.1 mmol/L. All subjects i) were between 18 and 60 years of age, ii) had not received medication within the past 3 months, iii) were non-alcohol abusers (alcohol consumption: < 140 g/week in men and < 70 g/week in women), and iv) exhibited menstrual regularity (women). Exclusion criteria were subjects who had i) moderate or severe hypertension, ii) moderate or severe renal and hepatic dysfunction, or iii) abnormal pituitary hormones. All subjects provided written informed consent and the study protocol had the approval of the local Ethical Committee of the First Affiliated Hospital of Chongqing Medical University (#2020-854). Clinical and biochemical parameters were measured as described previously.^14^

### Animals

Seven-week-old male C57BL/KsJ *db/db* mice and their wild-type littermates were from GemPharmatech (Nanjing, China). C57BL/6J mice (16 weeks old) were from Chongqing Medical University Animal Experimental Center. All animals were housed at a constant temperature (22 °C) with a fixed 12 h/12 h light/dark cycle and received a regular diet. At 16 weeks of age, the *db/db* mice developed significantly increased blood glucose and body weight compared with littermate controls (Table S1). They were sacrificed and their plasma, epididymal fat pads, liver, skeletal muscle, and pancreas were quickly collected. Explants of tissues were cultured as described previously.^14^ The University Animal Care Committee approved all procedures.

### Isolation of exosomes

Isolation of human plasma exosomes (1 mL/subject) and culture media exosomes (500 μL/well) was performed with commercial isolation kits (Total Exosome Isolation from plasma and Total Exosome Isolation from cell culture media, Invitrogen, USA). The quality and purity of the exosomes were identified as described previously.^14^

### Human plasma exosomal miRNA sequencing assay

Human plasma exosomal RNAs were isolated with a miRNeasy Micro Kit (QIAGEN, Germany). The quantity and quality of the extracted exosomal RNA were determined with an Agilent Bioanalyzer 2100 and a Small RNA Chip (Agilent Technologies, Santa Clara, CA, USA). Then, sequencing libraries were generated with a Next Multiplex Small RNA Library Prep Set for Illumina (NEB, USA) and index codes were added to attribute sequences to each sample. Libraries were sequenced with the Illumina HiSeq platform. For data analysis, the sequencing reads were aligned to the miRbase and Human Genome (GRCh38). Differential expression of miRNAs was undertaken with edgeR; only miRNA with Q-value <0.001 and Log_2_ fold change >1 was selected as candidate miRNA.

### Culture of Min6 cells, isolated mouse islets, and isolated human islets

Min6 cells were a gift from the Catholic University of Louvain, Belgium. The cells were cultured in Dulbecco's modified Eagle medium (Invitrogen, Shanghai, China) containing 10% fetal bovine serum (BI, Shanghai, China) and 1% penicillin/streptomycin (Beyotime Institute of Biotechnology, Shanghai, China) at 37 °C with 5% CO_2_. The mouse pancreas was cannulated with 2 mL cold Krebs–BSA 2% buffer (KRBB) containing collagenase P (1.3 mg/2 mL) (Roche Diagnostics, Mannheim, Germany) and digested as described previously.^14^ The primary mouse islets were cultured in RPMI-1640 medium (Invitrogen) containing 2 mM glutamine (Sigma–Aldrich, Brazil), 5 g/L bovine serum albumin (Sigma–Aldrich, New Zealand), and 1% penicillin/streptomycin at 37 °C with 5% CO_2_.

Between July 2022 and November 2023, primary human islets were isolated from human pancreatic tissues obtained from non-diabetic patients who had undergone partial pancreatectomy due to various pancreatic diseases (benign or malignant pancreatic tumors) (Table S2). The protocol had the approval of our university's local ethical committee (#2021-302). Soft and healthy pancreatic specimens (>2 g) were immersed in Belzer UW Cold Storage Solution (Brige to Life, Northbrook, USA) and delivered on ice to the laboratory. The pancreatic tissue was injected with a digestive enzyme solution (130 mL of RPMI-1640 media with 20 mL of 5 mg/mL Liberase RI; Roche Diagnostics) and then minced into ∼4 mm^3^ pieces, followed by incubation in a water bath at 37 °C for 10–15 min by manual shaking. After digestion, the pancreas pellets were washed three times in cold RPMI-1640 media. Single islets were manually selected under a microscope and cultured in a modified CMRL1066 medium (iCellTrans X018I0, China) supplemented with 0.5% human serum albumin (#S20170005, CSL Behring AG, Switzerland), 2 mM glutamine, and 1% penicillin/streptomycin at 37 °C with 5% CO_2_. The protocol was adapted from that of Botticher et al.^15^ With this method, we can obtain 20–80 human islets each time.

In some experiments, the cultured Min6 cells and islets were stimulated with or without glucose (Sigma–Aldrich, Shanghai, China), free fatty acids (FFAs) (0.067 mmol palmitic acid and 0.133 mmol oleic acid dissolved in 20 mL of 5% bovine serum albumin) (Sigma–Aldrich, Shanghai, China), and mixed cytokines [30 ng/mL INF-γ, 10 ng/mL TNFα, and 0.1 ng/mL IL-1β for mouse islets (R&D, USA); 100 U/mL INF-γ, 500 U/mL TNF-α, and 50 U/mL IL-1β for human islets (Cusabio, Wuhan, China)].

### Transfection of miR-146b mimics in Min6 cells, mouse islets, and human islets

Synthetic double-stranded oligonucleotides mimicking mature endogenous miR-146b (miR-mimic) and miR-mimic negative control (GeneCopoeia, Guangzhou, China) were delivered into Min6 cells or islets. Delivery was performed with Lipofectamine RNAiMAX transfection reagent (#13778075, Invitrogen, USA). The fluorescently labeled miR146b mimic was used to confirm the transfection efficiency. Overexpression of miR-146b was confirmed by real-time quantitative PCR (RT-qPCR) (Fig. S1). The medium was replaced with fresh medium 24 h post-transfection in all cases.

### Overexpression of Btg2 in Min6 cells

Min6 cells were transfected with a plasmid pReceiver-M02 containing the mouse *Btg2* sequence or a control plasmid containing a scrambled sequence (GeneCopoeia) with Lipofectamine 3000 Transfection Reagent (Invitrogen). Overexpression of *Btg2* was confirmed by RT-qPCR and western blotting (Fig. S2).

### Proliferation assays

The proliferation of Min6 cells was detected by the EdU method with fluorescent dye. Briefly, Min6 cells (40,000 cells/cm^2^) were seeded in a 24-well plate and incubated with miR-146b mimics for 72 h, and 10 μM EdU was added 12 h before the cells were fixed. Primary mouse (20/well) and human (2/well) islets were seeded onto Matrigel (#356231, Corning, NY, USA) coated coverslips to help their adherence and growth, and incubated with miR-146b mimics as well as 10 μM EdU for 72 h. EdU was detected with an EdU AlexaFluor 555 Imaging Kit (#C10338, Invitrogen, USA). Insulin was stained with an anti-insulin antibody (#ab181547, Abcam, Shanghai, China) and fluorescent secondary antibody (#4412, Cell Signaling Technology, Shanghai, China) to define beta-cells and islets. The images were observed and analyzed with a confocal fluorescence microscope (NIKON A1RHD25, Japan).

### Apoptosis assays

Min6 cells (40,000 cells/cm^2^) were seeded in a 24-well plate and incubated with miR-146b mimics for 72 h. Then, cells were collected and double-stained with fluorescein isothiocyanate–Annexin V and propidium iodide. The apoptosis rate of cells was detected with a flow cytometer (BD Bioscience, San Jose, CA, USA) and analyzed with CellQuest software (BD Biosciences). The apoptosis of primary mouse islets (20/well) and primary human islets (2/well) was measured with TUNEL (#T6013S, US EVERBRIGHT, China) and Annexin-V (#C1065S, Beyotime Institute of Biotechnology, Shanghai, China) with fluorescent dye. The images were observed and analyzed with a confocal fluorescence microscope (NIKON A1RHD25, Japan).

### Glucose-stimulated insulin secretion assays

Min6 cells (40,000 cells/cm^2^), 20 size-matched mouse islets, or two size-matched human islets per well were transfected with miR-146b mimics or negative control mimics and incubated for 72 h. Cells or islets were then starved at 37 °C for 1 h in KRBB with 2.8 mM glucose and subsequently incubated at 37 °C for 1 h in KRBB supplemented with 2.8 or 16.7 mM of glucose. The insulin levels in the supernatants of cultured mice and human islets were measured with ELISA kits (for mice, Thermo Fisher Scientific, MA, USA; for humans, Cusabio, Wuhan, China). For the islets, the values were normalized to the total islet protein content measured with a Pierce BCA Protein Assay Kit (Thermo Fisher Scientific).

### Real-time quantitative PCR

For miRNA quantification, RNA was isolated with a miRNeasy Micro Kit (#217084, QIAGEN, Germany). Total RNA (0.2 μg) was reverse-transcribed with the All-in-One miRNA cDNA Synthesis Kit (#QP116, GeneCopoeia, Guangzhou, China). Total RNA equivalents (2 ng) were amplified with PowerUp SYBR Green (#A25742, Thermo Fisher Scientific, MA, USA) using commercial miRNA-specific primers (GeneCopoeia). Human snRNA U6 and mouse snoRNA U68 were used as control primers (GeneCopoeia). For mRNA quantification, RNA was extracted with TRIzol (Invitrogen). RT-qPCR was performed with designed primers (Table S3), as described previously.^16^ The control primer was 18srRNA. Relative changes in the expression level of one specific gene are presented as 2^−ΔΔCt^.

### Western blotting and immunofluorescence assays

An extract of 20 μg proteins was dissolved in Laemmli buffer, subjected to sodium dodecyl sulfate–polyacrylamide gel electrophoresis under reducing and heat-denaturing conditions, and then transferred to a polyvinylidene fluoride membrane. Antibodies (Table S4) were used for immunodetection. Signals were obtained by enhanced chemiluminescence (Amersham Imager 600, GE, USA). For immunofluorescence, the slices were double-stained with anti-Btg2 and anti-Insulin antibodies (Table S4). The scanned images were analyzed using Aipathwell software (Servicebio®, Wuhan, China).

### Luciferase assays

The 293T cells were seeded into a 24-well plate. When the cell confluence reached 70%–80%, 293T cells were transiently co-transfected with 25 nM miR-146b mimics or negative controls together with 0.1 μg psi-CHECK2 vector reporter plasmids or psi-CHECK2/Btg2-3′–UTR wild-type or mutant reporter plasmids with Lipofectamine 3000. 36 h post-transfection, the Dual-Luciferase Reporter Assay System (#E1910, Promega, USA) was used to detect firefly and Renilla luciferase activities, and recorded with Lumat^3^ LB 9508 (Berthold Technologies, Germany).

### Statistical analysis

Statistical analysis was performed with SPSS Statistics (IBM, Armonk, NY, USA, version 20). Variables were presented as mean ± standard deviation or standard error of the mean. Means of continuous variables were compared by the unpaired *t*-test or Mann–Whitney test (when the data were not normally distributed), or one-way ANOVA followed by the Newman–Keuls test (three groups). The percentage differences between groups were compared by *χ*^2^ tests. The correlations of miRNA expression levels with the clinical variables were evaluated by Pearson analysis. The differences and associations were considered statistically significant at *p* < 0.05.

## Results

### Exosomal miRNA expression profiling in the circulation of subjects

Plasma exosomes from five subjects with non-diabetic obesity, as well as the age- and sex-matched five subjects with healthy weight (Table 1), were profiled by high-throughput miRNA sequencing. The expression of 83 miRNAs was differently regulated between the two groups (Q-value <0.001 and Log_2_ fold change >1). Among them, 38 miRNAs were up-regulated and 45 miRNAs were down-regulated (Fig. S3 and Table S5).

**Table 1:** Clinical characteristics of subjects.

	Normal (*n* = 25)	Obesity (*n* = 25)	Ob-T2DM (*n* = 20)	*p* values		
				Obesity *vs*. Normal	Ob-T2DM *vs*. Normal	Ob-T2DM *vs*. Obesity
Age (years)	29.6 ± 5.4	30.1 ± 6.7	30.0 ± 8.8	0.79	0.87	0.96
Male, n (%)	12 (48 %)	11 (44 %)	6 (30 %)	0.78	0.22	0.34
SBP (mmHg)	115.1 ± 14.0	129.3 ± 13.7	143.3 ± 23.7	<0.01	<0.01	0.02
DBP (mmHg)	72.9 ± 9.3	82.3 ± 7.4	91 ± 15.3	<0.01	<0.01	0.02
FPG (mmol/L)	5.4 ± 0.6	5.4 ± 0.4	9.3 ± 3.0	0.91	<0.01	<0.01
2hPG (mmol/L)	7.4 ± 2.2	7.5 ± 1.5	16.8 ± 4.3	0.88	<0.01	<0.01
HbA_1C_ (%)	5.4 ± 0.3	5.6 ± 0.3	8.5 ± 1.8	0.16	<0.01	<0.01
TC (mmol/L)	4.4 ± 0.7	4.4 ± 0.9	4.8 ± 0.6	0.88	0.15	0.17
TG (mmol/L)	1.0 ± 0.5	1.7 ± 0.9	2.3 ± 0.8	<0.01	<0.01	0.08
HDL (mmol/L)	1.7 ± 0.4	1.5 ± 0.7	1.0 ± 0.2	0.31	<0.01	0.01
LDL (mmol/L)	2.4 ± 0.9	2.4 ± 1.0	3.2 ± 0.6	0.97	0.01	0.02
Cr (μmol/L)	66.2 ± 12.4	66.1 ± 19.0	56.7 ± 14.2	0.99	0.09	0.39
UA (μmol/L)	294.1 ± 83.0	444.5 ± 149.6	393.1 ± 133.4	0.01	0.07	0.39
ALT (IU/L)	14.6 ± 5.7	55.8 ± 43.5	68.5 ± 46.6	<0.01	<0.01	0.45
AST (IU/L)	15.6 ± 4.6	33.5 ± 18.1	47.8 ± 29.8	<0.01	<0.01	0.12
BMI (kg/m^2^)	20.6 ± 1.9	31.7 ± 3.6	35.7 ± 6.4	<0.01	<0.01	0.01
WC (cm)	75.2 ± 8.0	103.2 ± 10.7	111.8 ± 13.7	<0.01	<0.01	0.03
FINS (uU/ml)	4.4 ± 1.4	16.3 ± 9.6	24.9 ± 13.5	<0.01	<0.01	<0.01
2hINS(uU/ml)	27.6 ± 13.2	111.5 ± 72.0	102.6 ± 72.5	<0.01	<0.01	0.68
HOMA-β	49.8 ± 19.3	176.0 ± 97.3	106.9 ± 76.8	<0.01	<0.01	0.01
HOMA-IR	1.0 ± 0.4	3.9 ± 2.5	10.0 ± 5.4	<0.01	<0.01	<0.01

Data are means ± SD or number (percentage) for the indicated number of subjects in each group. *p* values for comparisons between groups are based on an unpaired t-test or Mann–Whitney Test (when data was not normally distributed) or χ^2^ test. Insulin resistance and beta-cell secretory function are calculated using the following formulas: HOMA-IR = fasting insulin × fasting glucose/22.5; HOMA-β = 20 × fasting insulin/(fasting glucose-3.5). Normal = subjects with normal weight; Obesity = subjects with non-diabetic obesity; Ob-T2DM = subjects with both obesity and new-onset T2DM.

**Figure 1:**
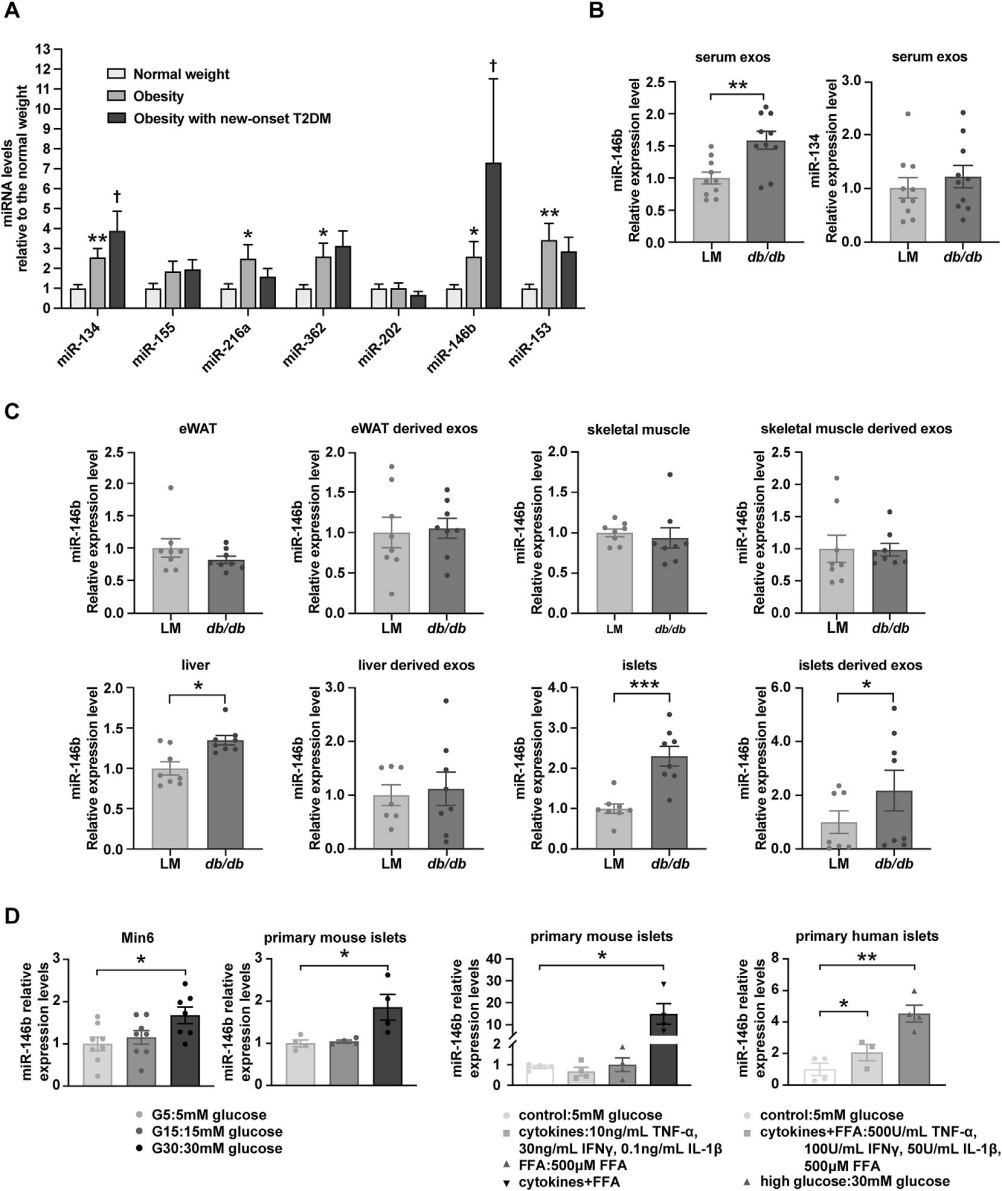
The exosomal miRNA expression in the circulation of the subjects, the tissue source of circulating up-regulated miR-146b, and the direct effects of high glucose, free fatty acids (FFAs), and inflammatory cytokines on islet miR-146b expression. **(A)** The seven miRNAs, which were significantly up-regulated (fold-change >2) and are known to be functional in cell survival, were further quantified by real-time quantitative PCR. Expression levels of these miRNAs were compared among three groups of subjects (*n* = 20 subjects/group). The values were presented as relative expression compared with healthy weight. Results are mean ± standard error of the mean (SEM). ∗*p* < 0.05, ∗∗*p* < 0.01, and ∗∗∗*p* < 0.001 versus healthy weight. ^+^*p* < 0.05 versus obesity. **(B, C)** Expression of miR-146b and miR-134 was quantified by real-time quantitative PCR in plasma exosomes, cultured tissue explants, and culture medium exosomes of *db/db* obese diabetic mice. The values were presented as relative expression compared with their wild-type littermates (LMs). Results are mean ± SEM for 8–10 mice/group. ∗*p* < 0.05, ∗∗*p* < 0.01, and ∗∗∗*p* < 0.001 versus respective LMs. **(D)** Min6 cells, primary mouse islets, and primary human islets were cultured with or without high glucose, FFAs, or inflammatory cytokines for 24 h. Expression of miR-146b was quantified by real-time quantitative PCR. The values were presented as relative expression compared with the control (5 mM glucose). Results are mean ± SEM for three experiments. ∗*p* < 0.05 and ∗∗*p* < 0.01 versus control.

We herein focused on investigating the miRNAs that were up-regulated by obesity. Among the 38 up-regulated miRNAs, we selected seven miRNAs (which had their predicted target genes with Targetscan and miRDB databases and have established roles in cell survival) for further verification. We quantified these seven miRNAs by RT-qPCR in another 20 subjects with non-diabetic obesity and 20 subjects with healthy weight, as well as 20 subjects with both obesity and new-onset T2DM (Table 1). We validated five out of the seven miRNAs by RT-qPCR as up-regulated by obesity. Interestingly, two miRNAs (miR-146b and miR-134) that were up-regulated in obesity were further up-regulated in new-onset T2DM (Fig. 1A). By correlation analysis in the overall cohort, we found that the expression of the two miRNAs was markedly positively correlated with the levels of fasting glucose and HbA_1C_ (Fig. S4). We further analyzed the independent associations between the miRNAs and the clinical variables in the separate groups. The two miRNAs displayed significantly positive associations with fasting plasma glucose only in the T2DM group after adjusting for age, sex, and body mass index (Table S6).

### Up-regulated expression of circulating miR-146b mainly originating from islets compared with other insulin-responsive tissues

Next, we determined the likely sources of the two elevated circulating miRNAs with the *db/db* mouse model. This model can mimic human obese T2DM because the *db/db* mice have leptin resistance, a remarkable feature of human obese T2DM. We first verified the expression of the two miRNAs in their plasma. The expression of miR-146b was up-regulated in the plasma of obese diabetic mice compared with their lean littermates, whereas the expression of miR-134 did not increase (Fig. 1B). Thus, we preferentially studied miR-146b. We detected the expression of miR-146b in the islets and other insulin-responsive tissues (including epididymal adipose tissue, skeletal muscle, and liver) and in their culture media. Interestingly, the expression of miR-146b increased slightly in the liver (∼1.3-fold) but increased obviously in islets (∼2.3-fold) and islet-secreted exosomes (∼2.2-fold) (Fig. 1C). By analyzing the GEO database, we also found that the expression of miR-146b did not show a noticeable change (>2-fold) in adipocytes, liver, and skeletal muscle (Fig. S5).

### Direct up-regulation of islet miR-146b by high glucose, FFAs, and inflammatory cytokines

To further confirm the possible cause of the change in miR-146b expression detected in the islets of *db/db* mice, we exposed Min6 cells and mouse islets to high glucose, considering the strong relationship between miR-146b and hyperglycemia in human T2DM status. As expected, the expression of miR-146b was enhanced in response to 30 mM glucose (Fig. 1D). Yet, miR-146a, another member of the miR-146 family and having been reported functional in beta-cells, was not changed under this stimulation (Fig. S6), consistent with a previous study.^17^

Given that increased FFAs and inflammation are the main features of obesity, we also treated mouse islets with high FFAs and inflammatory cytokines. Although miR-146b did not respond to either high FFAs or cytokines, it was markedly up-regulated by a mix of the two factors (Fig. 1D). We obtained similar results in human islets (Fig. 1D).

To further support our observation that the up-regulation of miR-146b mainly occurred in islets among the insulin-responsive tissues, we checked the miR-146b expression in mature 3T3-L1 adipocytes and LO2 hepatocytes treated with high glucose as well as FFAs. Conversely, miR-146b was or tended to be down-regulated in these cells (Fig. S7).

### The action of miR-146b on apoptosis of islet beta-cells

**Figure 2:**
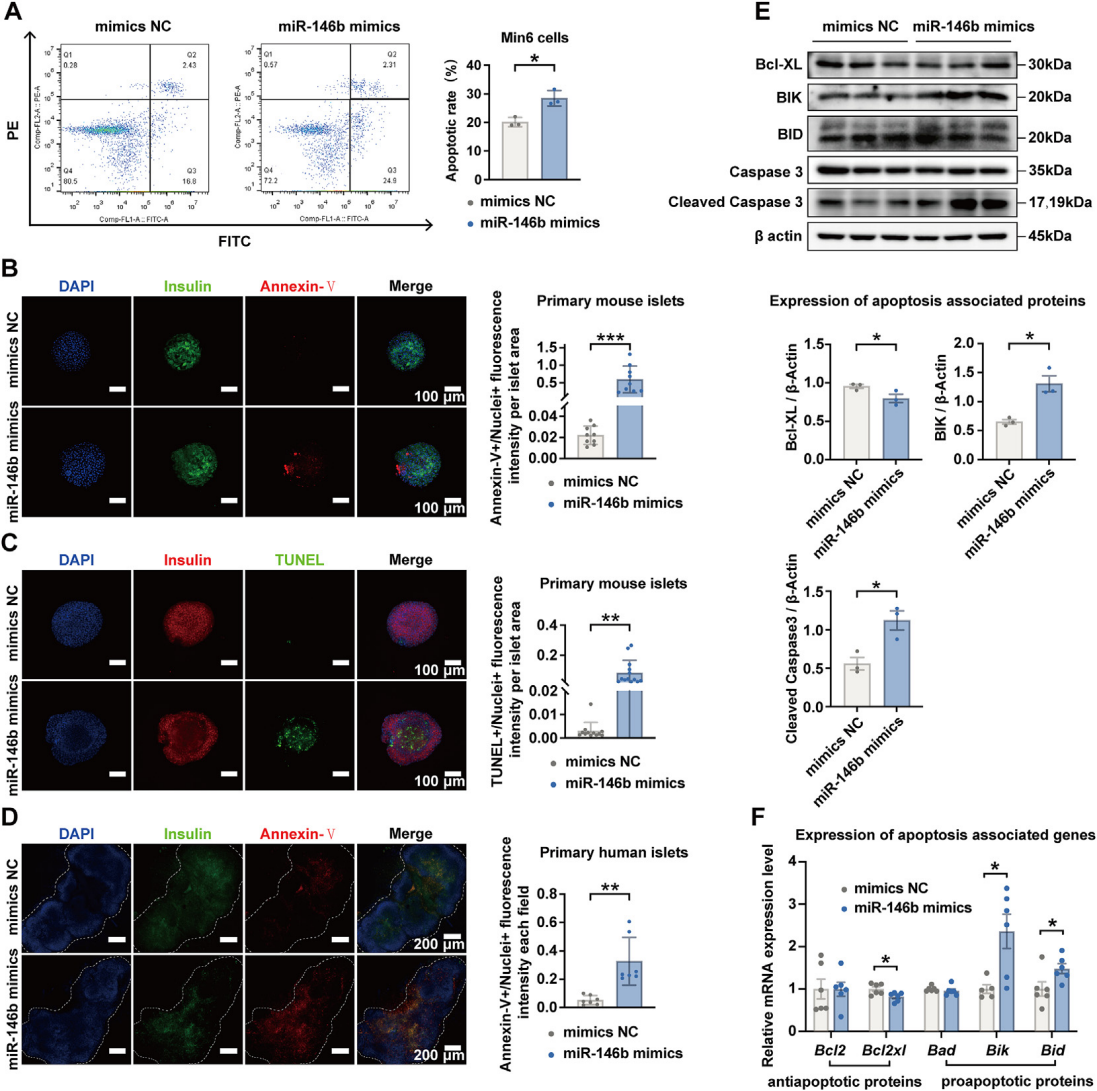
Effect of miR-146b overexpression on islet beta-cell apoptosis. **(A)** Min6 cells were transfected with 25 nM miR-146b mimics and incubated with annexin-V and propidium iodide for 72 h. The apoptosis of cells was detected with a flow cytometer. The data were presented as the apoptosis rate (apoptotic cell numbers/total cell numbers). **(B, C)** Primary mouse islets were transfected with 25 nM miR-146b mimics and incubated with annexin-V or TUNEL for 72 h. The data were presented as a ratio (annexin-V/TUNEL fluorescence intensity/nuclei fluorescence intensity per islet area). **(D)** Primary human islets were transfected with 25 nM miR-146b mimics and incubated with annexin-V for 72 h. The data were presented as a ratio (annexin-V fluorescence intensity/nuclei fluorescence intensity per islet area). **(E)** The abundance of apoptotic proteins in transfected Min6 cells was measured by western blotting. Representative blots are shown on the top. **(F)** The gene expression of Bcl family members in transfected Min6 cells was quantified by real-time quantitative PCR. In all the experiments, the cells or islets transfected with the miR-mimic negative control were taken as the controls (mimics NC). Results are mean ± standard error of the mean for three experiments. ∗*p* < 0.05 and ∗∗∗*p* < 0.001 versus mimics NC.

We observed that apoptosis of islet beta-cells was facilitated by miR-146b. In miR-146b-overexpressed Min6 cells, the number of apoptotic cells detected with a flow cytometer was much higher than that in control cells (Fig. 2A). For mouse islets, we used both annexin-V and TUNEL fluorescence staining to detect cellular apoptosis, which indicated increasing intensity with increasing annexin-V-positive cells or TUNEL-positive nuclei in miR-146b-overexpressed islets (Fig. 2B, C). We also observed stronger signals with increasing annexin-V-positive cells in miR-146b-overexpressed human islets (Fig. 2D). It is well-known that apoptosis manifests in two major execution programs, including the caspase pathway and Bcl family members, which are proteins with both proapoptotic and antiapoptotic properties. We found that overexpression of miR-146b increased the protein content of cleaved caspase 3 (an active form of caspase 3) (Fig. 2E) and increased the gene as well as protein expression of pro-apoptotic protein Bik, and yet decreased anti-apoptotic protein Bcl2xl, thereby modulating the balance toward apoptosis (Fig. 2E and F).

### The action of miR-146b on proliferation, insulin synthesis, and insulin secretion of islet beta-cells

**Figure 3:**
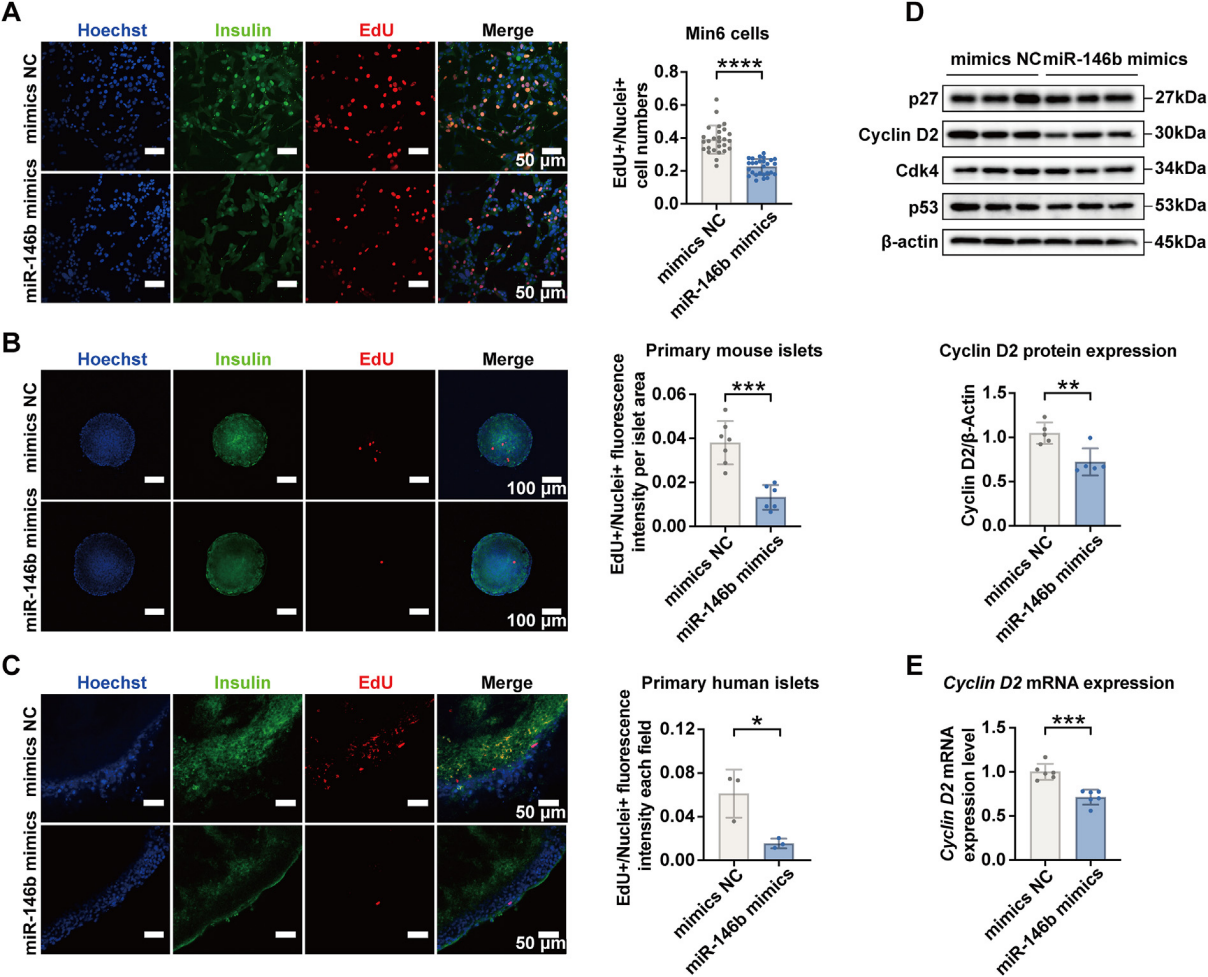
Effect of miR-146b overexpression on islet beta-cell proliferation. **(A)** Min6 cells were transfected with 25 nM miR-146b mimics for 72 h. EdU was added 12 h before cells were fixed and detected with a fluorescent dye. The data were presented as a ratio (EdU^+^ cell numbers/nuclei^+^ cell numbers). **(B, C)** Primary mouse islets and human islets were transfected with 25 nM miR-146b mimics and incubated with EdU for 72 h. The data were presented as a ratio (EdU fluorescence intensity/nuclei fluorescence intensity per islet area). **(D)** The abundance of cell cycle proteins in transfected Min6 cells was measured by western blotting. The representative blots are shown on the top. **(E)** The gene expression of cyclin D2 in transfected Min6 cells was quantified by real-time quantitative PCR. In all the experiments, the cells or islets transfected with the miR-mimic negative control were taken as the controls (mimics NC). Results are mean ± standard error of the mean for three experiments. ∗*p* < 0.05 and ∗∗∗*p* < 0.001 versus mimics NC.

The results of EdU fluorescence indicated that the proliferation of Min6 beta-cells, primary mouse islets, and human islets was reduced by miR-146b (Fig. 3A–C). To obtain insight into whether this suppression of beta-cell proliferation by miR-146b was affecting the cell cycle, we checked the expression of cell cycle activators such as Cdk4 and cyclin D2, as well as cell cycle inhibitors including p27 and p53. The protein and gene expression of cyclin D2 was significantly down-regulated as a result of miR-146b overexpression (Fig. 3D, E).

**Figure 4:**
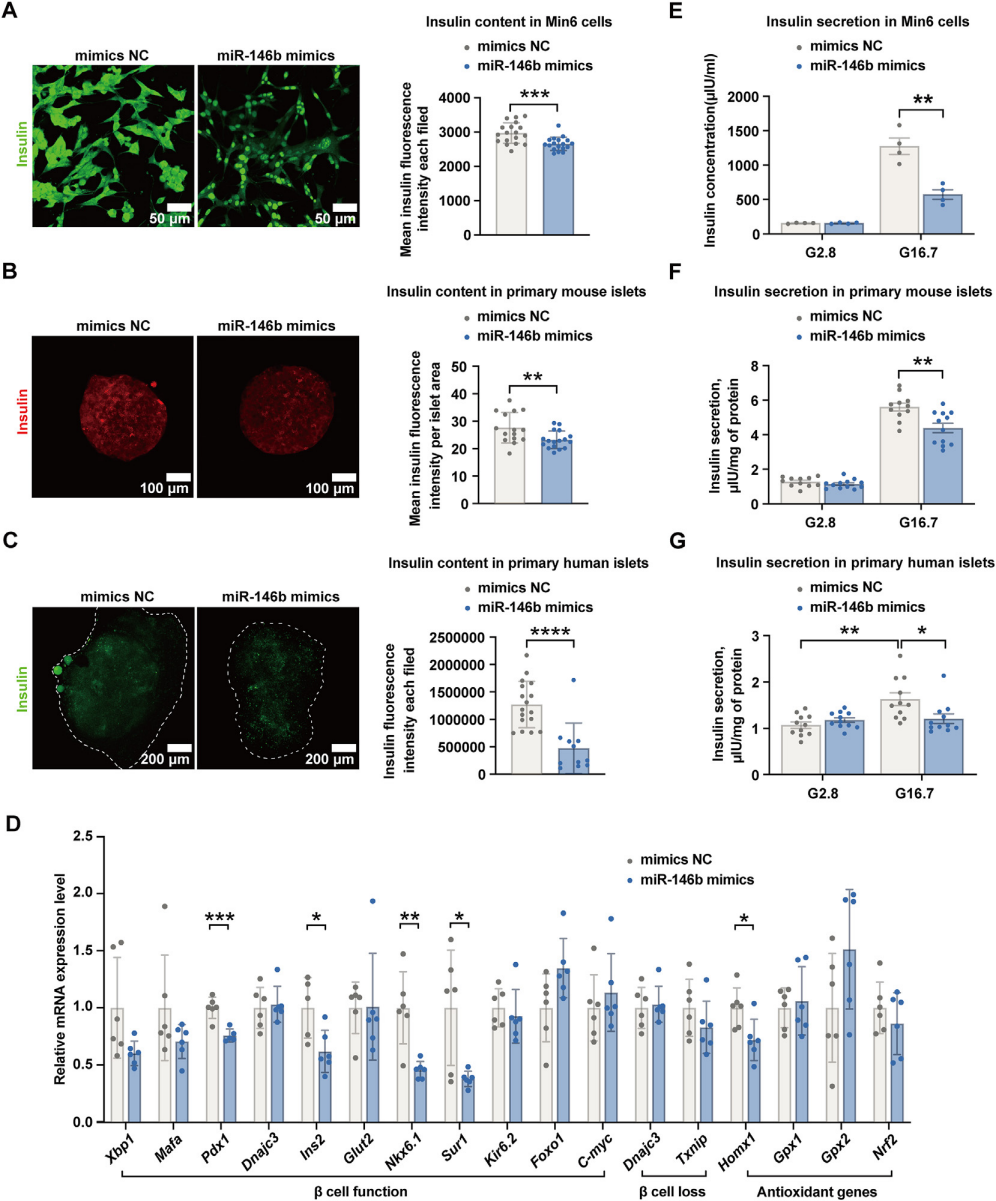
Effect of miR-146b overexpression on insulin synthesis and secretion. **(A**–**C)** Min6 cells, primary mouse islets, and human islets were transfected with 25 nM miR-146b. Seventy-two hours post-transfection, insulin was stained with an anti-insulin fluorescent antibody. The data were presented as mean insulin fluorescence intensity per field or islet area. **(D)** Expression of a series of transcription factors associated with insulin synthesis was quantified by real-time quantitative PCR in transfected Min6 cells. **(E**–**G)** Min6 cells, primary mouse islets, and primary human islets were transfected with 25 nM miR-146b mimics. Seventy-two hours post-transfection, the basal and glucose-stimulated insulin levels were measured by ELISA. For islets, the insulin levels were calibrated by total islet protein content. In all the experiments, the cells or islets transfected with the miR-mimic negative control were taken as the controls (mimics NC). Results are mean ± standard error of the mean for three experiments. ∗*p* < 0.05, ∗∗*p* < 0.01, and ∗∗∗*p* < 0.001 versus mimics NC.

Next, we investigated the role of miR-146b in insulin synthesis. We observed that miR-146b overexpression in Min6 cells, mouse islets, and human islets slightly but significantly decreased insulin contents (10%–15%) (Fig. 4A–C). Moreover, we found that the expression of a series of transcriptional factors associated with insulin synthesis was modified by miR-146b. Among them, the expression of *Pdx1*, *Ins2*, *Nkx6.1*, *Sur1*, and *Homx1* were significantly down-regulated in Min6 cells overexpressing miR-146b (Fig. 4D). Finally, we found that miR-146b overexpression blunted the high glucose-stimulated insulin secretion (Fig. 4E–G).

### Involvement of target genes of miR-146b in cell survival

**Figure 5:**
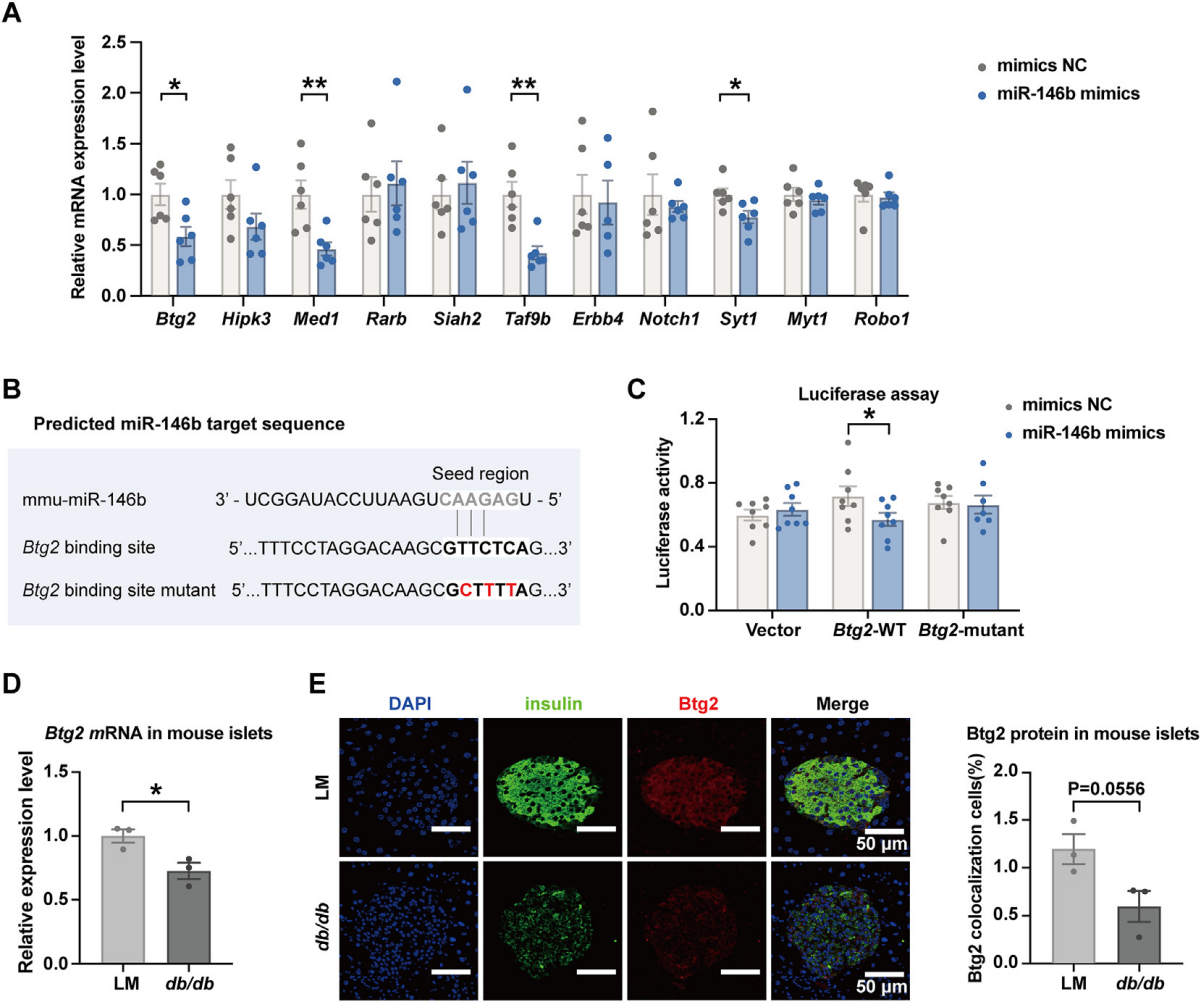
Identification of the predicted target genes of miR-146b *in vitro*. **(A)** The predicted target genes involved in the positive regulation of proliferation, negative regulation of proliferation, negative regulation of the apoptotic process, and cell differentiation were quantified by real-time quantitative PCR in Min6 cells transfected with miR-146b mimic or negative control (mimics NC). **(B)** The predicted binding site between miR-146b and its target gene *Btg2*. **(C)** Relative luciferase activity of Min6 cells co-transfected with miR-146b mimics and a luciferase reporter containing Btg2 binding sequence. The data were presented as the relative ratio of Renilla luciferase activity to firefly luciferase activity. Results are mean ± standard error of the mean for three experiments. ∗*p* < 0.05 versus mimics NC. **(D)** The gene expression of *Btg2* in the islets of *db/db* mice (6 mice/group) was quantified by real-time quantitative PCR. ∗*p* < 0.05 versus respective littermates (LMs). **(E)** The protein expression of *Btg2* in the islets of *db/db* mice was semi-quantified by immunofluorescent staining (3 slices/group). The data were presented as the ratio of colocalization cells [colocalization cells/(red cells + green cells − colocalization cells) × 100 %].

To further elucidate the functional effects of miR-146b, we evaluated the potential target genes of mouse miR-146b with Targetscan and miRDB databases. These two algorithms jointly predicted that 70 genes might be targeted by miR-146b. Based on annotation with the GO biological process, 24 genes correspond to the regulation of cellular proliferation, apoptosis, and differentiation (Fig. S8). We quantified the expression of parts of these genes by RT-qPCR in Min6 cells overexpressing miR-146b. Three genes (*Btg2*, *Med1*, and *Taf9b*) corresponding to the negative regulation of the apoptotic process and one gene (*Syt1*) corresponding to cell differentiation were suppressed by miR-146b (Fig. 5A). Next, we performed luciferase reporter assays to verify the direct binding between the four genes and miR-146b. We observed that only *Btg2* exhibited decreased activity in the presence of a miR-146b mimic, whereas this inhibitory effect of miR-146b was abolished when we mutated the binding site (Fig. 5B, C). The link between miR-146b and *Btg2* was also indicated by the fact that the gene and protein expression of *Btg2* were down-regulated in the islets of *db/db* mice (Fig. 5D, E), where the expression of miR-146b was up-regulated. Moreover, we utilized the same miRNA databases to predict the target genes of human miR-146b, we found that *Btg2* was also a target of human miR-146b, indicating its conservation between mouse and human.

**Figure 6:**
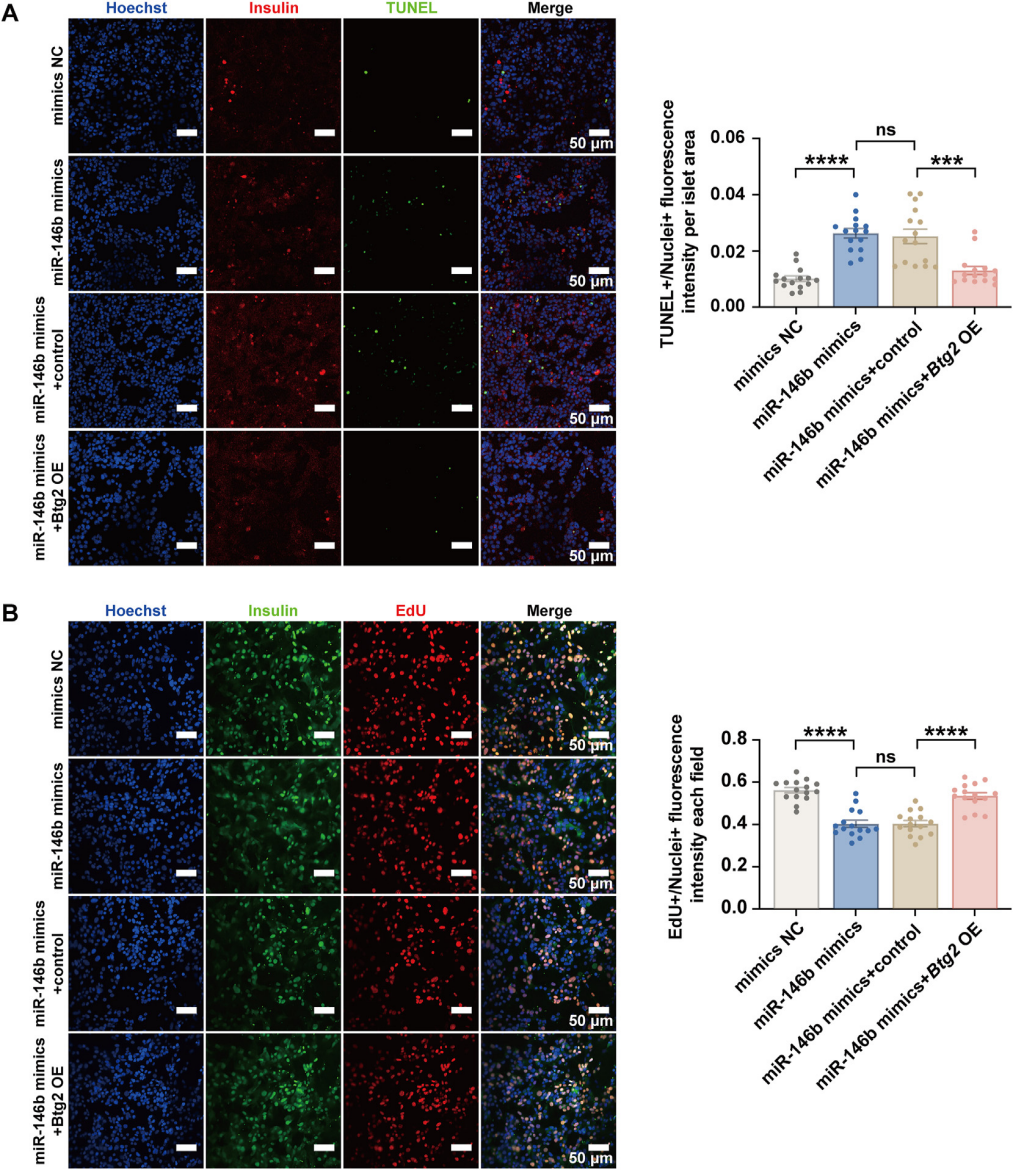
The rescue effect of *Btg2* overexpression in β-cells overexpressing miR-146b. Min6 cells were transfected with a plasmid containing the mouse *Btg2* sequence (*Btg2* OE) or a control plasmid (control) and co-transfected with miR-146b mimics or negative control. Seventy-two hours after transfection, apoptosis (by TUNEL) **(A)** and proliferation (by EdU) **(B)** were detected as described previously. Results are mean ± standard error of the mean for three experiments. ∗*p* < 0.05 and ∗∗∗*p* < 0.001 versus mimics NC or miR-146b mimics + control.

To verify whether the pro-apoptosis effect of miR-146b in beta-cells was mediated by *Btg2*, we performed a rescue experiment by overexpressing *Btg2* in Min6 cells. Our results showed that *Btg2* overexpression abolished miR-146b mimics-induced apoptosis (Fig. 6A) while restoring the suppressed proliferation (Fig. 6B).

## Discussion

In the present study, we investigated miRNAs involved in inducing beta-cell decline during obesity. We evaluated circulating exosomal miRNAs that are up-regulated in obesity and have been documented to play roles in cell survival. We found that miR-146b expression in obesity and T2DM exhibited a dynamic change. It was mildly up-regulated in non-diabetic obesity (∼2-fold versus healthy controls) and dramatically up-regulated further in newly established T2DM (∼7-fold versus healthy controls). Moreover, we found that this miRNA was up-regulated in islets of *db/db* diabetic mice and up-regulated in mouse as well as human islets under the direct stimulation of obesity- and T2DM-linked conditions (high FFAs, inflammatory cytokines, and high glucose). Notably, the miR-146 family (miR-146a and miR-146b) has been regarded as islet-enriched and diabetes-related miRNAs. Guo et al revealed that miR-146b expression in the circulation was dramatically augmented in children with obesity and augmented in adult T2DM patients. They also found that the serum levels of miR-146b were gradually up-regulated with the development of obesity and diabetes in *ob/ob* obese mice as well as *db/db* diabetic mice. Their results suggest that this miRNA has the potential to predict the risk of developing T2DM in children with obesity.^18^ In addition, Raitoharju et al found that levels of miR-146b in human circulation were positively associated with the levels of HbA_1C_.^19^ Conversely, expression of blood miR-146a was found by most studies to be reduced in diabetes and was likely related to an augmented inflammatory response.^20^^,^^21^

Although studies to date have revealed the relevance of altered levels of the miR-146 family with obesity and T2DM, its precise functions (especially in islet beta-cells) have seldom been investigated. One initial study revealed that elevated levels of miR-146a contributed to increased islet beta-cell apoptosis induced by FFAs.^17^ In subsequent research, blocking miR-146a function protected Min6 beta-cells from cytokine-triggered cell death.^22^ Regarding miR-146b, only one study by Guo et al reported that it could blunt insulin secretion in Min6 beta-cells after stimulation with high glucose.^18^ Nevertheless, in other cell systems (such as mouse chondrocytes, porcine ovarian granulosa cells, intestinal epithelial cells, and human dendritic cells), miR-146b facilitates cellular apoptosis.^23^, ^24^, ^25^, ^26^ In mouse and human adipocytes, miR-146b inhibits cellular proliferation and decreases insulin sensitivity.^18^^,^^27^ To our knowledge, our study is the first to investigate the roles of miR-146b in beta-cell survival. We found that miR-146b facilitated apoptosis yet suppressed proliferation by affecting the key regulatory molecules in these two pathways. Moreover, it decreased insulin synthesis by modifying a series of related transcriptional factors. These abnormalities may jointly contribute to impaired high glucose-stimulated insulin secretion. Our results indicate that miR-146b could be a potential negative beta-cell number and function regulator. Thus, the modest increase of miR-146b during obesity (increased FFAs and chronic inflammation) might cause inadequate beta-cell compensation, leading to increased blood glucose levels. The increased blood glucose, in turn, further up-regulated the expression of miR-146b. This vicious circle might contribute to the progression toward T2DM (Fig. S9).

Since one miRNA can have numerous targets, the mechanism through which miR-146b can affect beta-cell survival must be elucidated. At present, several targets of miR-146b have been evaluated in other cell systems, such as hepatocytes, monocytes, and chondrocytes. In these cells, miR146b targets interleukin-1 receptor-associated kinase 1 (IRAK1) and tumor necrosis factor receptor-associated factor 6 (TRAF6), two key components of Toll-like receptor signaling that mediate activation of the NF-κB pathway.^28^^,^^29^ In our study, we verified for the first time that in beta-cells, miR-146b can directly target the *Btg2* gene, which could protect beta-cells from apoptosis and facilitate proliferation. Physiological roles of *Btg2* have been suggested as prodifferentiative and anti-apoptotic transcriptional factors in both mice and humans.^30^, ^31^, ^32^ It has been reported that *Btg2* expression increases during neurogenesis whereas deletion of this gene facilitates programmed cell death.^33^ Interestingly, *Btg2* is up-regulated in expanded rodent islets during pregnancy, suggesting its involvement in the adaptive growth of beta-cells.^34^ Thus, down-regulation of *Btg2* by miR-146b could contribute to beta-cell decline. It would be interesting to identify other targets of miR-146b by combining RNA-sequencing and miRNA databases in future experiments.

In conclusion, our findings add more evidence for the potential of miR-146b as a diagnostic biomarker for T2DM risk in adults with obesity. Longitudinal studies are needed to further investigate this miRNA's diagnostic efficiency. In addition, our findings reveal the functional roles of islet miR-146b, which targets *Btg2* and is involved in negatively regulating beta-cell number and function. These findings provide new insights into the molecular mechanisms predisposing beta-cells to decline. Controlling the miR-146b/Btg2 axis might facilitate new T2DM prevention and management therapeutic strategies.

## CRediT authorship contribution statement

**Weixuan Wang:** Conceptualization, Data curation, Visualization, Writing – original draft. **Dan Ma:** Data curation. **Yong Chen:** Resources. **Rui Cheng:** Methodology, Writing – original draft, Writing – review & editing. **Ting Zhang:** Methodology. **Qian Ge:** Conceptualization, Funding acquisition, Writing – original draft, Writing – review & editing. **Xi Li:** Funding acquisition, Writing – review & editing.

## Data availability

The datasets generated during and/or analyzed during the current study are available from the corresponding author upon reasonable request.

## Funding

This work was supported by the 10.13039/501100005230Natural Science Foundation of Chongqing, China (No. CSTC2020jcyj-msxmX0093 to Qian Ge), the National Key R&D Program of China (No. 2018YFA0800401 to Xi Li), the National Natural Science Foundation of China (No. 81770861, 82070899 to Xi Li), the general project of the Natural Sciences Foundation of Chongqing, China (No. CSTB2022NSCQ-MSX0827 to Xi Li), and the CQMU Program for Youth Innovation in Future Medicine (Chongqing, China) (No. W0046 to Xi Li).

## Conflict of interests

No conflict of interests needs to be declared.
