# Supplementary material for: miR-146b/Btg2 axis as a potential inducer of islet beta-cell decline during the progression of obesity to T2DM
Source: Genes Dis. 2025 Apr 2;12(5):101621. doi: 10.1016/j.gendis.2025.101621 (PMC12242404; doi:10.1016/j.gendis.2025.101621)
Supplement: Multimedia component 1 [file mmc1.docx]

Supplementary Table 1

| Mouse (16 week) | Weight (g) | Plasma glucose (mmol/L) |
| --- | --- | --- |
| db/db-1 | 63.7 | 26.1 |
| db/db-2 | 58.9 | 28.1 |
| db/db-3 | 71.9 | 33.3 |
| db/db-4 | 62.2 | 22.7 |
| db/db-5 | 66.5 | 33.3 |
| db/db-6 | 66.0 | 29.7 |
| db/db-7 | 64.5 | 23.7 |
| db/db-8 | 67.5 | 30.1 |
| db/db-9 | 69.4 | 29.3 |
| db/db-10 | 53.8 | 28.3 |
| LM-1 | 29.9 | 7.9 |
| LM-2 | 30.1 | 8.5 |
| LM-3 | 27.0 | 7.5 |
| LM-4 | 26.4 | 8.7 |
| LM-5 | 26.0 | 7.3 |
| LM-6 | 28.2 | 9.2 |
| LM-7 | 28.6 | 7.3 |
| LM-8 | 29.3 | 6.2 |
| LM-9 | 29.5 | 9.4 |
| LM-10 | 31.4 | 10.2 |

LM= wild-type littermates
