# Supplementary material for: miR-146b/Btg2 axis as a potential inducer of islet beta-cell decline during the progression of obesity to T2DM
Source: Genes Dis. 2025 Apr 2;12(5):101621. doi: 10.1016/j.gendis.2025.101621 (PMC12242404; doi:10.1016/j.gendis.2025.101621)
Supplement: Multimedia component 2 [file mmc2.docx]

| Subjects | Sex | Age (year) | W (kg) | H (cm) | BMI (kg/m^2^) | DBP (mmHg) | SBP (mmHg) | ALT (IU/L) | AST (IU/L) | FPG (mmol/L) | Cr (μmol/L) |
| --- | --- | --- | --- | --- | --- | --- | --- | --- | --- | --- | --- |
| S1 | M | 64 | 84 | 164 | 31.2 | 134 | 87 | 32 | 25 | 6.8 | 72 |
| S2 | F | 48 | 54 | 165 | 19.8 | 105 | 68 | 15 | 35 | 4.6 | 51 |
| S3 | M | 58 | 52 | 153 | 22.2 | 153 | 90 | 14 | 20 | 7.1 | 29 |
| S4 | F | 50 | 55 | 148 | 25.1 | 114 | 76 | 12 | 18 | 5.1 | 51 |
| S5 | F | 59 | 64 | 158 | 25.6 | 138 | 89 | 10 | 12 | 5.4 | 49 |
| S6 | F | 31 | 56 | 155 | 23.3 | 114 | 66 | 17 | 21 | 6 | 49 |
| S7 | M | 54 | 46.5 | 165 | 17.1 | 112 | 73 | 101 | 56 | 5.1 | 55 |
| S8 | M | 32 | 80 | 172 | 27.0 | 123 | 81 | 12 | 17 | 5.4 | 79 |
| S9 | M | 65 | 60 | 170 | 20.8 | 160 | 98 | 95 | 55 | 4.9 | 72 |
| S10 | M | 57 | 65 | 167 | 23.3 | 163 | 109 | 70 | 50 | 6.4 | 66 |
| S11 | M | 49 | 65 | 170 | 22.5 | 140 | 91 | 74 | 86 | 5.7 | 67 |
| S12 | F | 55 | 60 | 150 | 26.7 | 145 | 72 | 29 | 37 | 6.1 | 35 |
| S13 | M | 66 | 48 | 170 | 16.6 | 87 | 61 | 39 | 51 | 5.1 | 33 |
| S14 | M | 53 | 56 | 163 | 17.6 | 125 | 83 | 67 | 60 | 5.8 | 56 |

**Supplementary Table 2. Characteristics of patients undergone partial pancreatectomy**

Data are means ± SD. Abbreviations: SBP=Systolic blood pressure; DBP=diastolic blood pressure, BMI=body mass index, FPG=fasting plasma glucose, ALT= alanine transaminase, AST= aspartate transaminase, Cr=creatinine
