# Supplementary material for: miR-146b/Btg2 axis as a potential inducer of islet beta-cell decline during the progression of obesity to T2DM
Source: Genes Dis. 2025 Apr 2;12(5):101621. doi: 10.1016/j.gendis.2025.101621 (PMC12242404; doi:10.1016/j.gendis.2025.101621)
Supplement: Multimedia component 3 [file mmc3.docx]

Supplementary Table 3. Gene sequences used as forward and reverse primers for RT-qPCR

| **Gene** | **Forward Primer** | **Reverse Primer** |
| --- | --- | --- |
| **Med1** | GATCCAGCCAGTCCAAAA | AAGGGTTCATAAGGGAGGA |
| **Btg2** | TTGAAGCCGTGTCTCGT | GGTCCTCTCCATCTTAGCC |
| **Erbb4** | TCCCCCAGGCTTTCAACATAC | GCTGTGTCCAATTTCACTCCTA |
| **Siah2** | TTTGACTATGTCCTGCCCCC | TTCCTGATGCTGGGCGTTAG |
| **Rarb** | AGGTGCCGAACGTGTAAT | GAACTTGGGGTCAAGGGT |
| **Taf9b** | CTTTGGTCACTACGGCTTG | TCCCTGTTGGCCTGATAG |
| **Hipk3** | CATCGAAGACAGCAAGCA | TGATGGGTATGGAAGCAGA |
| **Notch1** | GTGTTTATGGGCCTTGGG | GATGCTGTTTGGAGGGATG |
| **Syt1** | CTGCTGGTGGGAATCATC | CCGGTGGACTTTTGTCTC |
| **Myt1** | AGTGATGGCAGTGAGGATG | GCTGTCTGTGGGTGATGA |
| **Robo1** | AAGATGTCCCACCAACCA | CACTTCGACTGAGGAGGAA |
| **Bcl2** | AAACCCTCCATCCTGTCC | TCCTAAACCCTGCTTCCC |
| **Bcl2xl** | CCACAGGGTCAAGGGACT | CTAGGTGGGGCAGAGCA |
| **Bad** | CCCCTCCCAGTGATCTTCT | CGTCAGCTCTTCTCCCTGT |
| **Bik** | TGCTCCCTTCCTCTCTGC | TGCCTGCAAACACCACTT |
| **Bid** | CCCTCCGCTTCTGTATTTC | CATGGCTGGGATGAGTTC |
| **CyclinD2** | CCGTCCTTGGCTCTGGT | AGGCACCTGTTGAAACTGA |
| **Xbp-1** | AAACAGAGTAGCAGCGCAGACTGC | GGATCTCTAAAACTAGAGGCTTGGTG |
| **Mafa** | CAGCGGCACATTCTGGA | CCCGCCAACTTCTCGTATT |
| **Pdx-1** | CGGACATCTCCCCATACG | AAAGGGAGCTGGACGCGG |
| **Ins2** | ACCCACCCAGGCTTTTG | CCCAGCTCCAGTTGTTCC |
| **Glut2** | CATTCTTTGGTGGGTGGC | CCTGAGTGTGTTTGGAGCG |
| **Nkx6.1** | GGACCAGAGAGAGCACGC | TTCGGGTCCAGAGGTTTG |
| **Sur1** | CCCTAGCTGTGGTGTGCTACTTCA | GGGGCTGCGTTGTGTCATC |
| **Kir6.2** | TCGTGTCCAAGAAAGGCAACTG | GGAAGGCAGATGAAAAGGAGTGG |
| **Foxo1** | GTACAGCGCTAGCACCA | GCGACAGACAGAGTTCCC |
| **C-myc** | GTCTTCCCCTACCCGCTC | CTGTCCAACTTGGCCCTC |
| **Dnajc3** | AAGCCCGTGGAAGCCATTAG | GGTCATTTTCATTGTGCTCCTGAG |
| **Txnip** | CGGCTTTCGTTTTTCTTGAACC | TGACGGCTTTGACTCGGGTAAC |
| **Homx1** | CCACACAGCACTATGTAAAGCGTC | GTTCGGGAAGGTAAAAAAAGCC |
| **Gpx1** | ACAGTCCACCGTGTATGCCTTC | CTCTTCATTCTTGCCATTCTCCTG |
| **Gpx2** | TGATTGAGAATGTGGCGTCACTC | TTTGGGTAAGACTAAAGGTGGGC |
| **Nrf2** | CAGAGAAAAGGGAGAAAACGACAG | GGGAACAAGGAACACATTGCC |
| **18s** | CGCCGCTAGAGGTGAAATTCT | CATTCTTGGCAAATGCTTTCG |
