# Supplementary material for: miR-146b/Btg2 axis as a potential inducer of islet beta-cell decline during the progression of obesity to T2DM
Source: Genes Dis. 2025 Apr 2;12(5):101621. doi: 10.1016/j.gendis.2025.101621 (PMC12242404; doi:10.1016/j.gendis.2025.101621)
Supplement: Multimedia component 4 [file mmc4.docx]

Supplementary Table 4. Antibodies for Western blot (WB) or Immunofluorescence (IF)

| Antibody name | Dilution | Catalog # | Company |
| --- | --- | --- | --- |
| Rabbit anti-mouse β-Actin | 1:1000 (WB) | 4967L | [Cell Signaling Technology](https://www.baidu.com/link?url=fF_7z1S-MTxwotQOl-CXljiPRXvFyBEGLWSJeRIx-PoSmjIm58OmOvSKXDwzRHMW&wd=&eqid=ad1a69250007982c000000065dae776a), Shanghai, China |
| Rabbit anti-mouse Cyclin D2 | 1:1000 (WB) | 3741T | [Cell Signaling Technology](https://www.baidu.com/link?url=fF_7z1S-MTxwotQOl-CXljiPRXvFyBEGLWSJeRIx-PoSmjIm58OmOvSKXDwzRHMW&wd=&eqid=ad1a69250007982c000000065dae776a), Shanghai, China |
| Mouse anti-mouse CDK4 | 1:1000 (WB) | 2906 | [Cell Signaling Technology](https://www.baidu.com/link?url=fF_7z1S-MTxwotQOl-CXljiPRXvFyBEGLWSJeRIx-PoSmjIm58OmOvSKXDwzRHMW&wd=&eqid=ad1a69250007982c000000065dae776a), Shanghai, China |
| Rabbit anti-mouse p27 | 1:1000 (WB) | 3688S | [Cell Signaling Technology](https://www.baidu.com/link?url=fF_7z1S-MTxwotQOl-CXljiPRXvFyBEGLWSJeRIx-PoSmjIm58OmOvSKXDwzRHMW&wd=&eqid=ad1a69250007982c000000065dae776a), Shanghai, China |
| Mouse anti-mouse p53 | 1:1000 (WB) | SC-393031 | [Santa Cruz Technology](https://www.baidu.com/link?url=fF_7z1S-MTxwotQOl-CXljiPRXvFyBEGLWSJeRIx-PoSmjIm58OmOvSKXDwzRHMW&wd=&eqid=ad1a69250007982c000000065dae776a), Shanghai, China |
| Rabbit anti-mouse Bcl-XL | 1:1000 (WB) | 10783-1-AP | [Protein](https://www.baidu.com/link?url=fF_7z1S-MTxwotQOl-CXljiPRXvFyBEGLWSJeRIx-PoSmjIm58OmOvSKXDwzRHMW&wd=&eqid=ad1a69250007982c000000065dae776a)tech, Wuhan, China |
| Rabbit anti-mouse BIK | 1:1000 (WB) | PA5-20249 | Thermo Fisher Scientific, MA, US |
| Rabbit anti-mouse BID | 1:1000 (WB) | PA1-26454 | Thermo Fisher Scientific, MA, US |
| Rabbit anti-mouse Caspase-3 | 1:1000 (WB) | 9662S | [Cell Signaling Technology](https://www.baidu.com/link?url=fF_7z1S-MTxwotQOl-CXljiPRXvFyBEGLWSJeRIx-PoSmjIm58OmOvSKXDwzRHMW&wd=&eqid=ad1a69250007982c000000065dae776a), Shanghai, China |
| Rabbit anti-mouse Cleaved Caspase-3 | 1:1000 (WB) | 9661S | [Cell Signaling Technology](https://www.baidu.com/link?url=fF_7z1S-MTxwotQOl-CXljiPRXvFyBEGLWSJeRIx-PoSmjIm58OmOvSKXDwzRHMW&wd=&eqid=ad1a69250007982c000000065dae776a), Shanghai, China |
| Rabbit anti-mouse Btg2 | 1:1000 (WB)  1:500 (IF) | 22339-1-AP | Proteintech, Wuhan, China |
| Rabbit anti-mouse Insulin | 1:500 (IF) | GB12334 | Servicebio, Wuhan, China |
| Cy3 conjugated Goat Anti-Rabbit IgG (H+L) | 1:300 (IF) | GB21303 | Servicebio, Wuhan, China |
| Alexa Fluor® 488-conjugated Goat Anti-Mouse IgG (H+L) | 1:400 (IF) | GB25301 | Servicebio, Wuhan, China |

WB=Western blot, IF=Immunofluorescence
