# Supplementary material for: miR-146b/Btg2 axis as a potential inducer of islet beta-cell decline during the progression of obesity to T2DM
Source: Genes Dis. 2025 Apr 2;12(5):101621. doi: 10.1016/j.gendis.2025.101621 (PMC12242404; doi:10.1016/j.gendis.2025.101621)
Supplement: Multimedia component 5 [file mmc5.docx]

Supplementary Table 5. Differentially expressed miRNAs in subjects with normal weight and subjects with non-diabetic obesity

| Gene ID | log2 (Obese / Lean) | Qvalue [(Obese / Lean)] |
| --- | --- | --- |
| hsa-miR-31-3p | -10.07547915 | 0.017690615 |
| hsa-miR-550b-3p | 10.98584194 | 6.01E-10 |
| hsa-miR-4659b-3p | -12.84960057 | 0.022124085 |
| hsa-miR-3679-5p | 12.51224664 | 7.02E-12 |
| hsa-miR-6805-5p | -10.82336724 | 2.06E-11 |
| hsa-miR-134-5p | 4.93143278 | 0.015690088 |
| hsa-miR-27a-5p | -7.457538732 | 0.03859743 |
| hsa-miR-3619-3p | -12.31004468 | 8.15E-12 |
| hsa-miR-744-3p | -10.87728413 | 2.06E-11 |
| hsa-miR-155-5p | 3.012299265 | 0.036816766 |
| hsa-miR-3190-5p | 11.53867395 | 8.15E-12 |
| hsa-miR-216a-5p | 8.379378367 | 0.035581967 |
| hsa-miR-4738-3p | -11.23002044 | 1.09E-10 |
| hsa-miR-5010-5p | 13.09226162 | 3.20E-12 |
| hsa-miR-4772-3p | -10.35424938 | 4.53E-11 |
| hsa-miR-6736-5p | -10.9505559 | 1.99E-11 |
| hsa-miR-218-5p | -4.077025124 | 1.57E-04 |
| hsa-miR-133a-3p | -4.95419631 | 0.023226677 |
| hsa-miR-6821-5p | 11.14974712 | 4.33E-10 |
| hsa-miR-9-3p | 4.66500889 | 0.001106059 |
| hsa-miR-31-5p | -5.983361421 | 5.15E-04 |
| hsa-miR-1843 | -10.30378075 | 4.86E-11 |
| hsa-miR-302a-3p | -13.5372184 | 0.00449065 |
| hsa-miR-3613-5p | 3.117887736 | 0.022124085 |
| hsa-miR-4508 | -10.9076418 | 4.83E-12 |
| hsa-miR-885-3p | -11.79928162 | 1.52E-11 |
| novel-hsa-miR154-5p | 11.24792751 | 4.42E-11 |
| novel-hsa-miR148-3p | 5.105094676 | 0.006165118 |
| hsa-miR-214-5p | -11.25915477 | 0.001391834 |
| novel-hsa-miR130-3p | -10.48280796 | 3.96E-11 |
| novel-hsa-miR24-3p | -12.32558655 | 8.15E-12 |
| novel-hsa-miR293-5p | 6.283749836 | 0.0088439 |
| novel-hsa-miR187-5p | 11.83999107 | 9.61E-12 |
| novel-hsa-miR18-3p | -10.84705735 | 2.06E-11 |
| novel-hsa-miR172-5p | 10.34096276 | 4.30E-11 |
| hsa-miR-6715a-3p | 12.25207404 | 4.42E-11 |
| novel-hsa-miR90-5p | 11.73513288 | 1.56E-11 |
| novel-hsa-miR22-3p | -9.076815597 | 0.010292877 |
| novel-hsa-miR84-3p | 7.268520341 | 0.023522679 |
| novel-hsa-miR17-3p | 11.66044165 | 3.53E-11 |
| hsa-miR-378f | 4.364918236 | 0.001348752 |
| novel-hsa-miR54-5p | -11.30833903 | 9.21E-11 |
| hsa-miR-199b-3p | -5.985246233 | 0.004181808 |
| hsa-miR-4435 | -9.833048196 | 0.015311486 |
| hsa-miR-7976 | 12.43983088 | 3.40E-10 |
| hsa-miR-195-5p | 1.924898545 | 0.025311447 |
| hsa-miR-184 | -10.60779264 | 0.024120814 |
| hsa-miR-302c-3p | -12.48205163 | 0.0088439 |
| hsa-miR-550a-3-5p | -12.97799537 | 1.32E-11 |
| hsa-miR-362-5p | 6.291554446 | 0.03048743 |
| hsa-miR-615-3p | -11.39124359 | 0.011601726 |
| hsa-miR-195-3p | 11.2997804 | 9.61E-12 |
| hsa-miR-5585-3p | -10.85408918 | 4.83E-12 |
| hsa-miR-1290 | 5.337303321 | 0.032947564 |
| hsa-miR-5698 | 12.46454575 | 2.06E-11 |
| hsa-miR-365b-5p | -10.86805085 | 9.61E-12 |
| hsa-miR-1537-3p | 12.14625045 | 1.56E-11 |
| hsa-miR-140-3p | -7.900402396 | 0.010292877 |
| hsa-miR-4436b-5p | -10.64475759 | 1.22E-11 |
| hsa-miR-369-5p | -6.046676091 | 0.001632545 |
| hsa-miR-378b | 2.989742095 | 0.0088439 |
| hsa-miR-548t-5p | 12.07581338 | 9.61E-12 |
| hsa-miR-146b-5p | 2.476534996 | 0.035581967 |
| hsa-miR-153-3p | 2.681693011 | 0.0267846 |
| hsa-miR-122-3p | -7.974193944 | 0.033081567 |
| hsa-miR-1307-3p | 1.740407713 | 0.023226677 |
| hsa-miR-4725-3p | -11.26209485 | 1.00E-10 |
| hsa-miR-200c-3p | -8.987579838 | 0.002369804 |
| hsa-miR-769-5p | 6.561959681 | 0.00703422 |
| hsa-miR-943 | -11.24436384 | 3.20E-12 |
| hsa-miR-202-5p | 8.509906932 | 0.036816766 |
| hsa-miR-2278 | 7.876744304 | 0.03752692 |
| hsa-miR-3677-3p | -10.74903138 | 2.33E-11 |
| hsa-miR-302b-3p | -13.89888476 | 3.04E-04 |
| hsa-miR-92b-5p | -9.332122641 | 1.52E-04 |
| hsa-miR-302d-3p | -8.78312417 | 0.025981731 |
| hsa-miR-6885-3p | -10.37177664 | 4.42E-11 |
| hsa-miR-4516 | 7.573827491 | 0.011601726 |
| hsa-miR-449a | -8.998449397 | 3.44E-05 |
| hsa-miR-4525 | 12.82057788 | 1.50E-10 |
| hsa-miR-4466 | 14.51791539 | 2.66E-15 |
| hsa-miR-199a-3p | -5.985246233 | 0.004181808 |
| hsa-miR-496 | -10.77066389 | 0.033081567 |
