# Supplementary material for: miR-146b/Btg2 axis as a potential inducer of islet beta-cell decline during the progression of obesity to T2DM
Source: Genes Dis. 2025 Apr 2;12(5):101621. doi: 10.1016/j.gendis.2025.101621 (PMC12242404; doi:10.1016/j.gendis.2025.101621)
Supplement: Multimedia component 6 [file mmc6.doc]

|  | Normal | | Obesity | | Ob-T2DM | |
| --- | --- | --- | --- | --- | --- | --- |
|  | Beta* | *P* | Beta* | *P* | Beta* | *P* |
| miR-146b | | | | | | |
| FPG (mmol/L) | 0.04 | 0.88 | 0.24 | 0.39 | **0.72** | **<0.01** |
| HbA_1C_ (%) | 0.13 | 0.68 | 0.72 | 0.29 | 0.90 | 0.06 |
| TG (mmol/L) | -0.19 | 0.55 | 0.51 | 0.26 | 0.36 | 0.44 |
| FINS (uU/ml) | -0.13 | 0.58 | 0.17 | 0.53 | -0.51 | 0.18 |
| miR-134 | | | | | | |
| FPG (mmol/L) | 0.18 | 0.45 | 0.09 | 0.70 | **0.74** | **<0.01** |
| HbA_1C_ (%) | 0.58 | 0.01 | -0.10 | 0.86 | 0.68 | 0.18 |
| TG (mmol/L) | 0.05 | 0.89 | 0.32 | 0.26 | 0.00 | 1.00 |
| FINS (uU/ml) | 0.17 | 0.38 | 0.32 | 0.14 | -0.45 | 0.26 |

Supplementary Table 6. Associations between the miRNAs (miR-146b and miR-134) and the clinical variables in the separate groups.

The associations are analyzed using multiple linear regression. *The Standardized Coefficients were adjusted for age, sex, and BMI. Normal=subjects with healthy weight; Obesity=subjects with non-diabetic obesity; Ob-T2DM=subjects with both obesity and new-onset T2DM.
