# Supplementary material for: miR-146b/Btg2 axis as a potential inducer of islet beta-cell decline during the progression of obesity to T2DM
Source: Genes Dis. 2025 Apr 2;12(5):101621. doi: 10.1016/j.gendis.2025.101621 (PMC12242404; doi:10.1016/j.gendis.2025.101621)
Supplement: Multimedia component 7 [file mmc7.pdf]

Supplement Figure.1

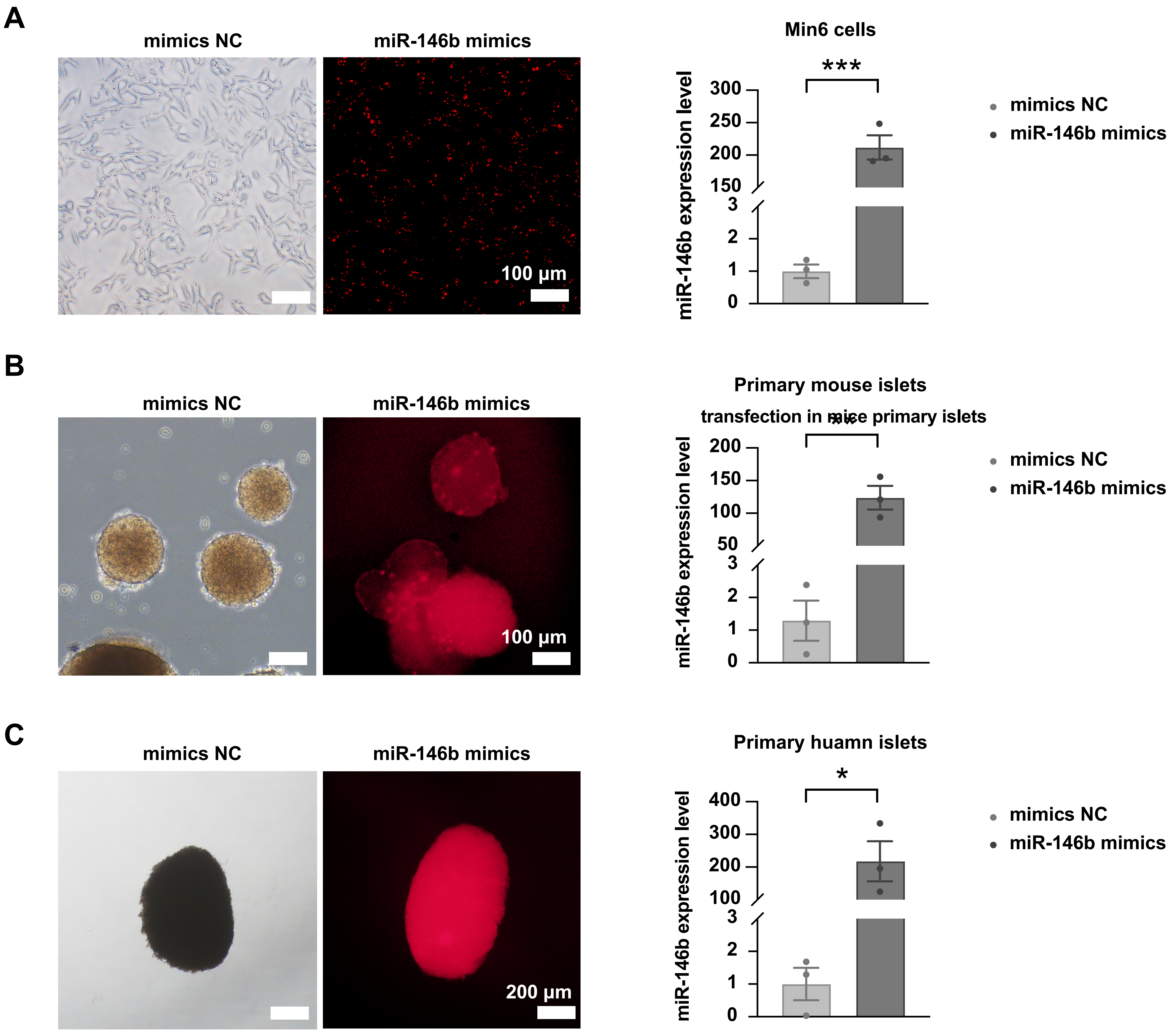

Supplement Figure.2

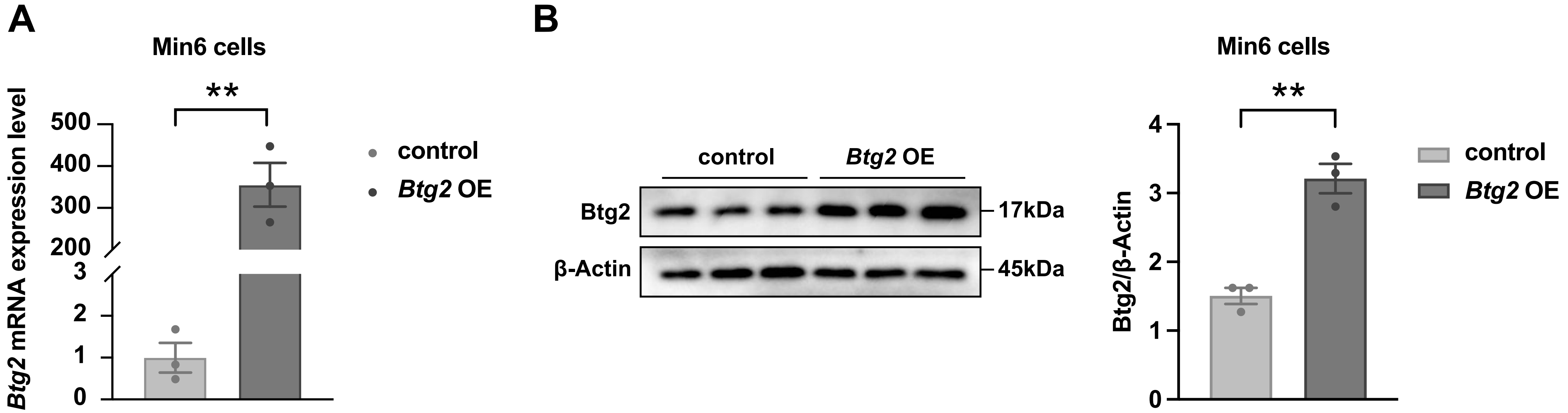

Supplement Figure.3

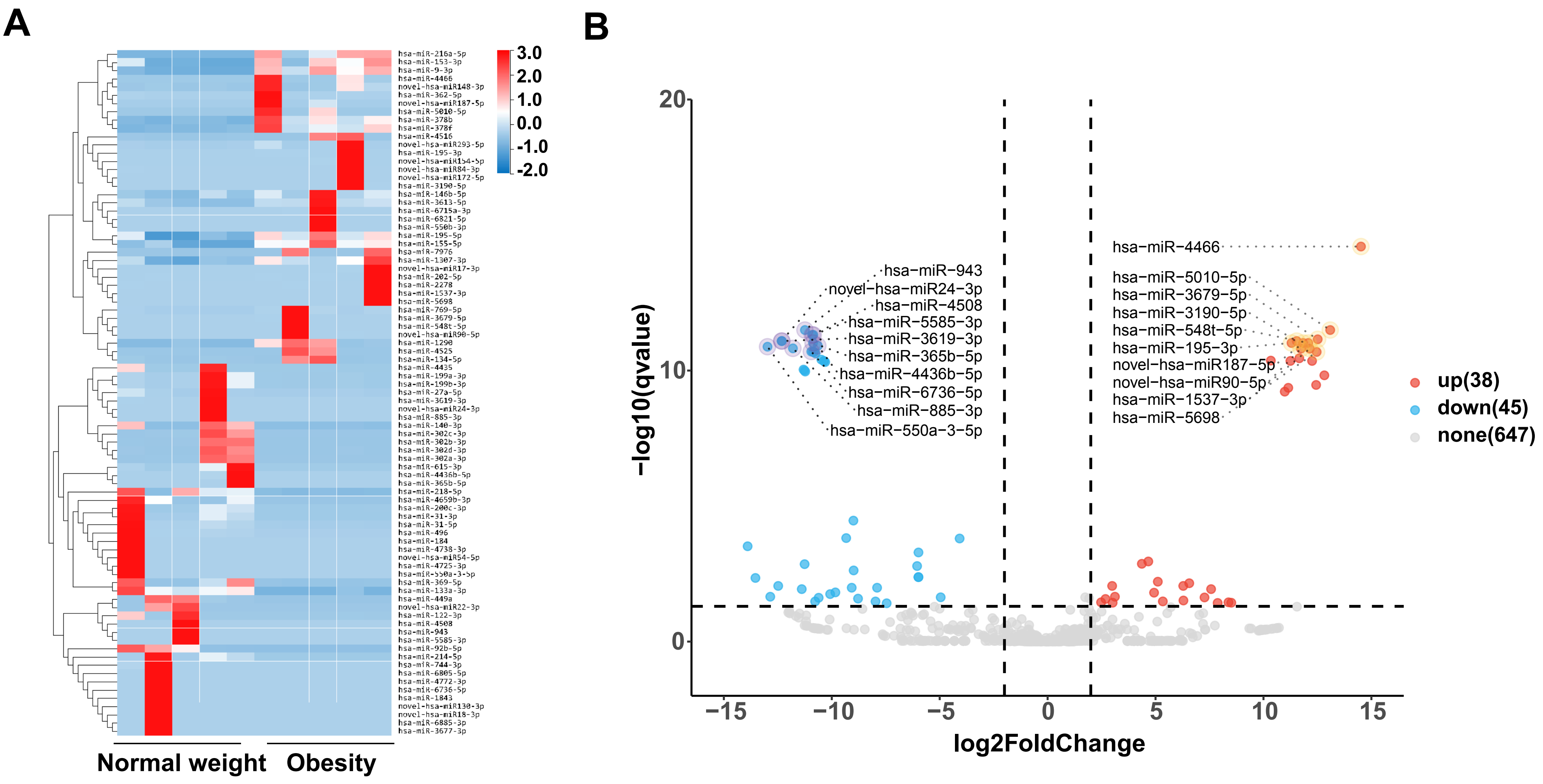

Supplement Figure.4

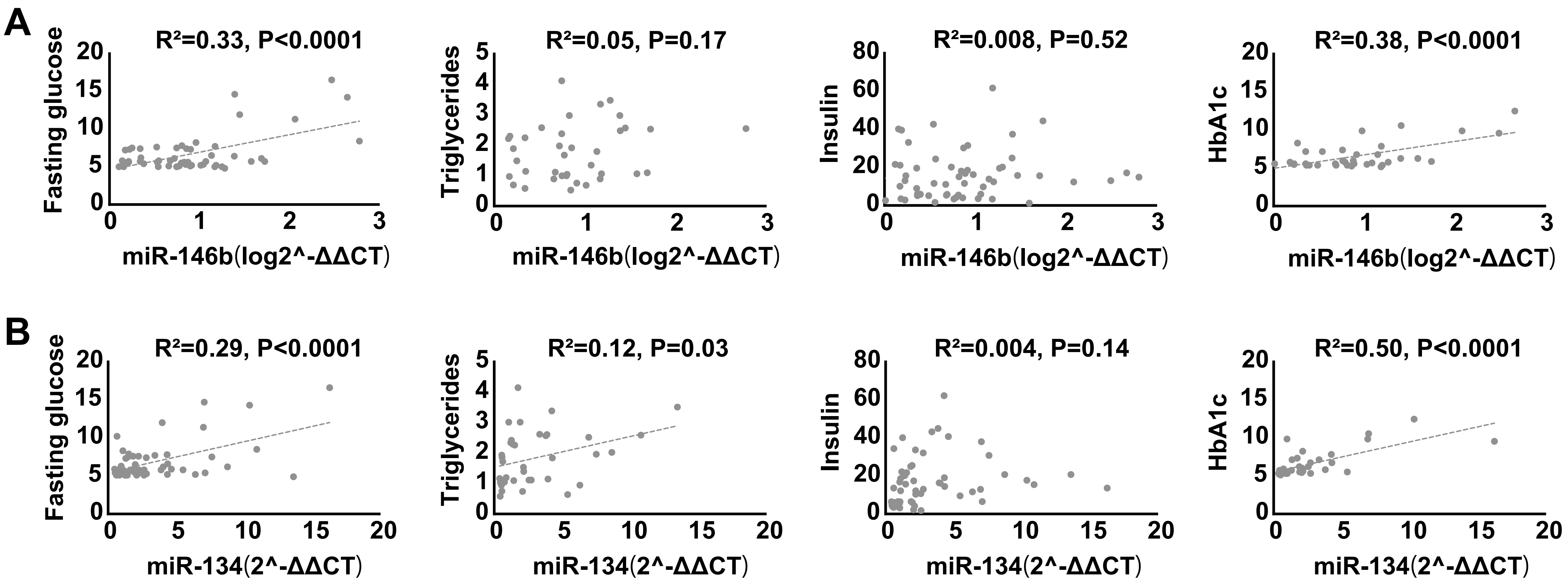

Supplement Figure.5

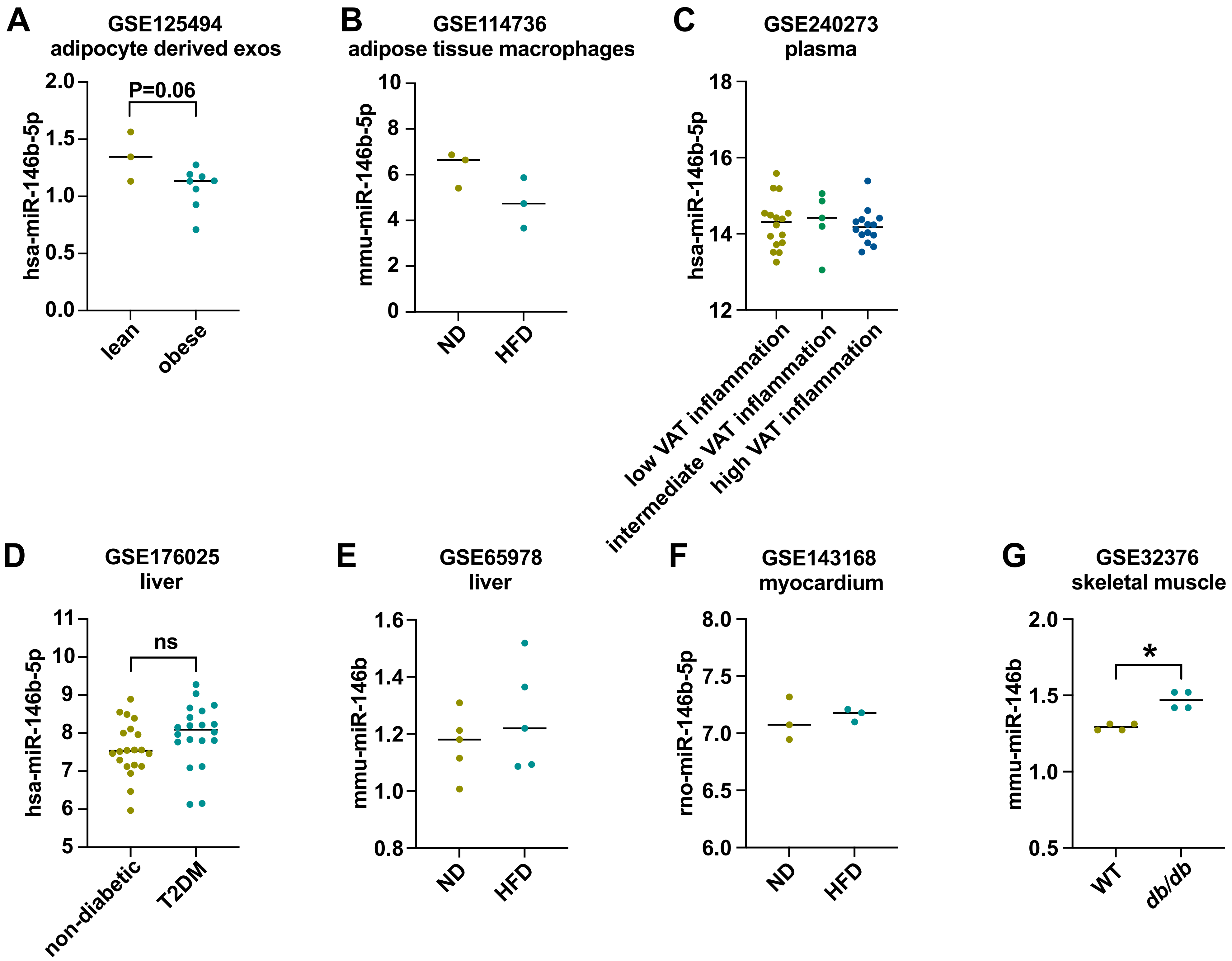

Supplement Figure.6

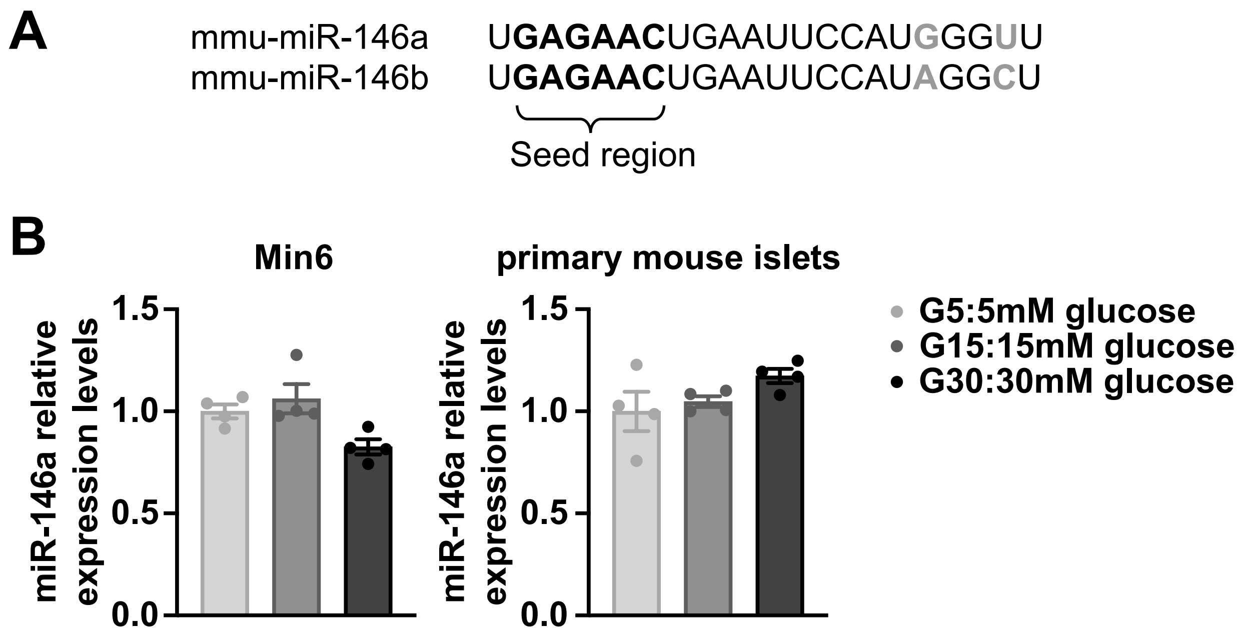

Supplement Figure.7

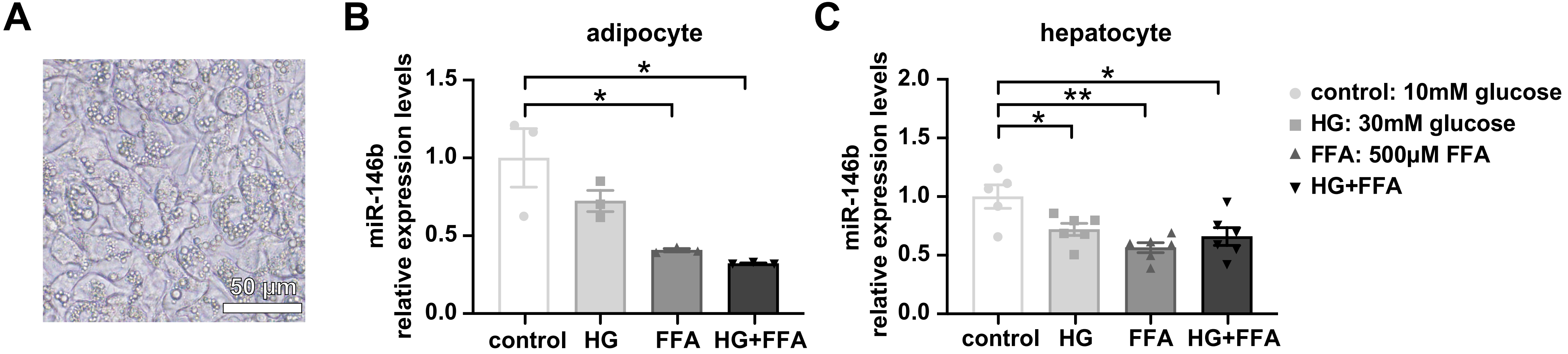

Supplement Figure.8

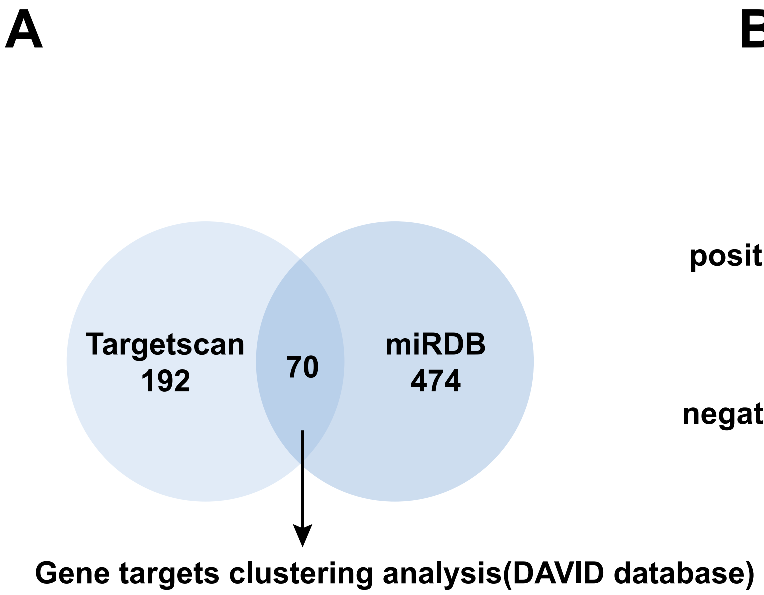

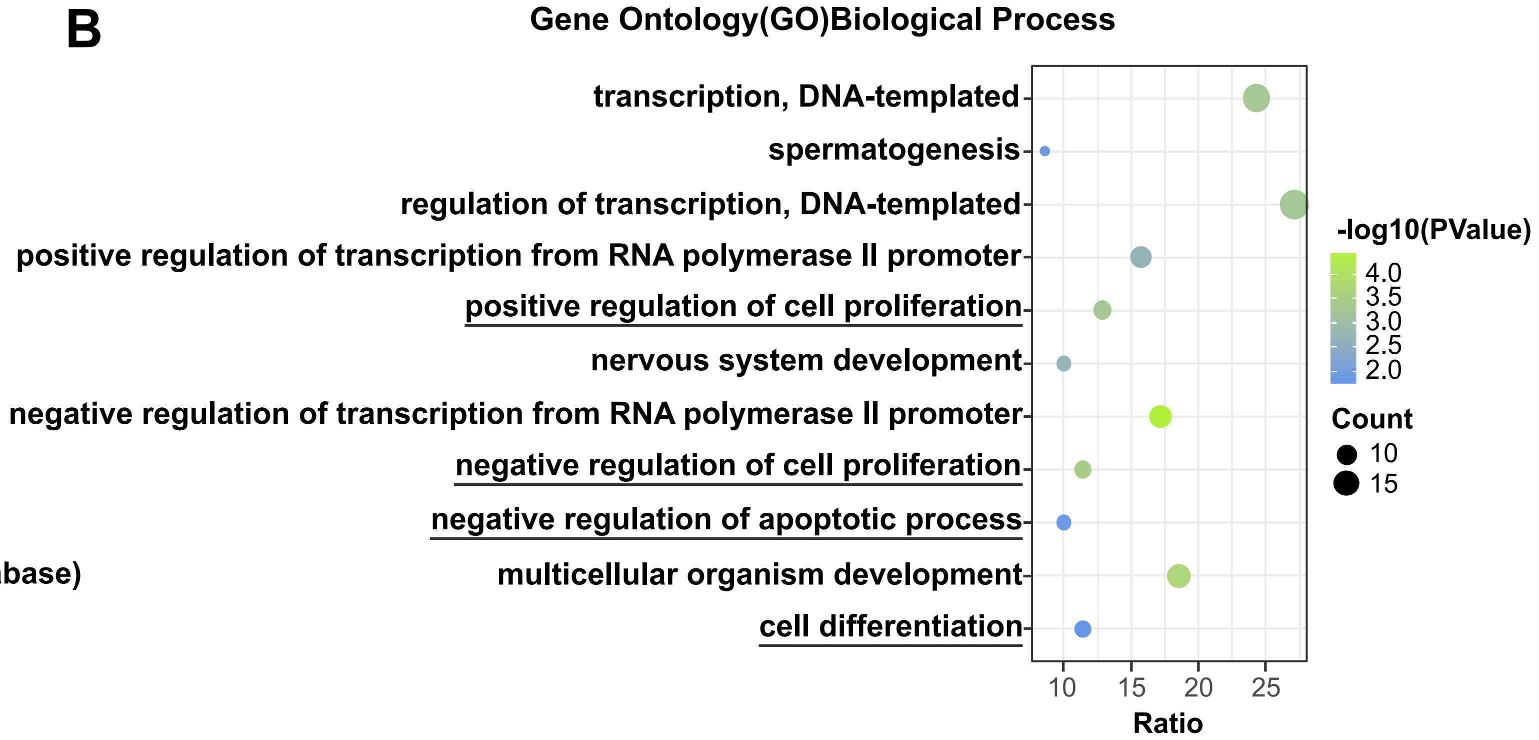

Supplement Figure.9

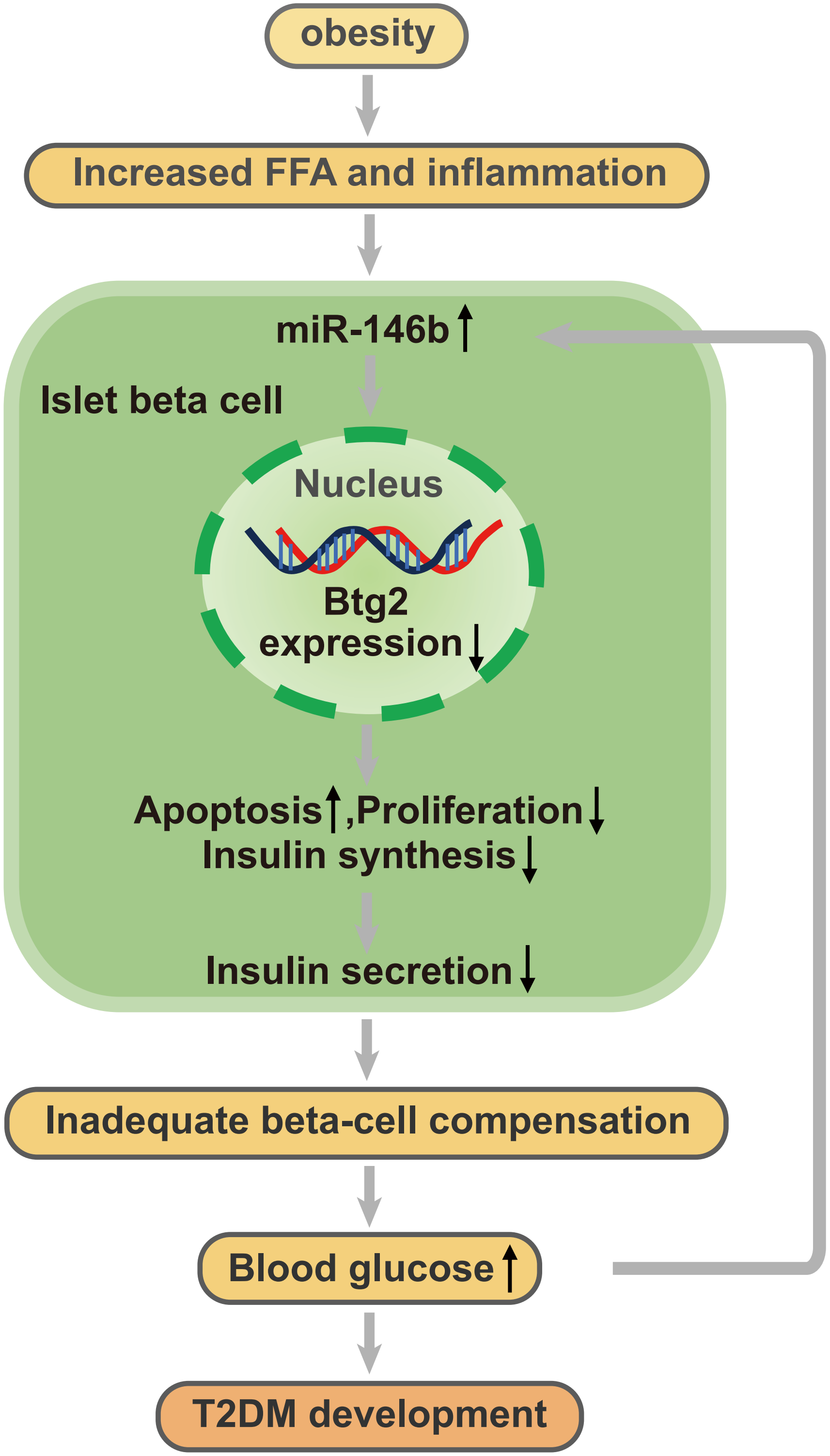

LEGENDS TO SUPPLEMENTARY FIGURES

Supplementary Figure 1. Verification of the overexpression of miR-146b in Min6 cells, primary mouse islets, and primary human islets. The Min6 cells **(A)**, primary mouse islets **(B)**, and primary human islets **(C)** were transfected with 25nM fluorescently labeled miR-146b mimics or negative control (mimics NC). The overexpression of miR-146b was confirmed by the red fluorescence and expression levels quantified by RT-qPCR.

Supplementary Figure 2. Verification of the overexpression of *Btg2* in Min6 cells. The Min6 cells were transfected with 1ng/ul *Btg2*-expressing plasmid (Btg2 OE) or control plasmid (control). The expression levels of *Btg2* gene **(A)** and protein **(B)** were quantified by RT-qPCR and Western blot respectively.

Supplementary Figure 3. The differentially expressed miRNAs between subjects with healthy weight and subjects with non-diabetic obesity. (A) The heat map shows the clustering of differential expressed miRNAs between subjects with normal weight and subjects with non-diabetic obesity. Red indicates a high relative expression; blue indicates a low relative expression. n=5 subjects/group. **(B)** The Volcano plot shows that 83 miRNAs were differently expressed between the two groups, 38 miRNAs were up-regulated, and 45 miRNAs were down-regulated, and 647 miRNAs were unchanged. The marked miRNAs were the top 10 up- and down-regulated miRNAs.

Supplementary Figure 4. Correlations between miRNA expression levels and clinical metabolic variables. miR-146b and miR-134, which were up-regulated in obesity and further up-regulated in obese T2DM, were analyzed. The miRNA expression levels are presented as $2^{-\Delta\Delta Ct}$, the correlations of which with clinical variables were evaluated by Pearson analysis. As the values of miR-146b were not normally distributed, the log transformation was used.

Supplementary Figure 5. Gene Expression Omnibus (GEO) database analysis of miR-146b expression in different tissues. The data were downloaded and analyzed from the online GEO database (https://www.ncbi.nlm.nih.gov/geo/).

Supplementary Figure 6. The expression of miR-146a under the stimulation of glucose. (A) Sequences of miR-146b and miR-146a. **(B)** Min6 cells and primary mouse islets were cultured with 5 mM (control), 15 mM, and 30 mM glucose for 24 h. Expression of miR-146a was quantified by RT-qPCR. Results are means ± SEM for 3 experiments.

Supplementary Figure 7. The expression of miR-146b in adipocytes and hepatocytes under the stimulation of glucose and free fatty acid (FFA). (A, B) The 3T3-L1 cells (a gift from Fudan University) were cultured in a DMEM medium containing 1% biotin. Two days after confluence (d 0), adipocyte differentiation was initiated by adding the addition of 1μg/ml insulin, 1μM dexamethasone, and 0.5 mM isobutylmethylxanthine for 48 h. Then, cells were refed by basal medium containing 1μg/ml insulin. On d 4, more than 70% of cells had accumulated fat droplets as shown on the left. These cells were cultured with or without high glucose or/and FFA from d 6-7. **(C)** The LO2 hepatocytes (Institute for Viral Hepatitis, Chongqing Medical University) were cultured in RPMI-1640 medium with or without high glucose or/and FFA for 24 h. Expression of miR-146b was quantified by RT-qPCR. Results are means ± SEM for 3 experiments. *P<0.05, **P<0.01 *vs.* control.

Supplementary Figure 8. The prediction of target genes of miR-146b. (A) The target genes of miRNA were predicted using the miRanda and Targetscan. The genes overlapped in the results of the two tools were taken as the target genes. **(B)** The biological function of target genes was annotated using the DAVID database (https://david.ncifcrf.gov).

Supplementary Figure 9. Scheme illustrating for the involvement of miR-146b during the progression of obesity to T2DM.
